# Dual-Omics Approach Unveils Novel Perspective on the Quality Control of Genetically Engineered Exosomes

**DOI:** 10.3390/pharmaceutics16060824

**Published:** 2024-06-18

**Authors:** Christopher Olson, Konstantin Ivanov, Darin Boyes, David Bengford, Joy Ku, Renceh Flojo, Pengyang Zhang, Biao Lu

**Affiliations:** 1 Department of Bioengineering, Santa Clara University, 500 El Camino Real, Santa Clara, CA 95053, USA; colson2@alumni.scu.edu (C.O.); kivanov@scu.edu (K.I.); dbengford@alumni.scu.edu (D.B.); jku@scu.edu (J.K.); rflojo@alumni.scu.edu (R.F.);; 2 Department of Biology, Santa Clara University, 500 El Camino Real, Santa Clara, CA 95053, USA; dboyes@alumni.scu.edu

**Keywords:** extracellular vesicles, exosome, proteomics, transcriptomics, quality control

## Abstract

Exosomes, nanoscale vesicles derived from human cells, offer great promise for targeted drug delivery. However, their inherent diversity and genetic modifications present challenges in terms of ensuring quality in clinical use. To explore solutions, we employed advanced gene fusion and transfection techniques in human 293T cells to generate two distinct sets of genetically engineered samples. We used dual-omics analysis, combining transcriptomics and proteomics, to comprehensively assess exosome quality by comparing with controls. Transcriptomic profiling showed increased levels of engineering scaffolds in the modified groups, confirming the success of genetic manipulation. Through transcriptomic analysis, we identified 15 RNA species, including 2008 miRNAs and 13,897 mRNAs, loaded onto exosomes, with no significant differences in miRNA or mRNA levels between the control and engineered exosomes. Proteomics analysis identified changes introduced through genetic engineering and over 1330 endogenous exosome-associated proteins, indicating the complex nature of the samples. Further pathway analysis showed enrichment in a small subset of cellular signaling pathways, aiding in our understanding of the potential biological impacts on recipient cells. Detection of over 100 cow proteins highlighted the effectiveness of LC-MS for identifying potential contaminants. Our findings establish a dual-omics framework for the quality control of engineered exosome products, facilitating their clinical translation and therapeutic applications in nanomedicine.

## 1. Introduction

In recent years, the field of nanomedicine has witnessed a remarkable surge in the use of human cell-derived extracellular vesicles, particularly exosomes [1,2,3,4]. These nano-scale, lipid-encapsulated vesicles (30~150 nm) have emerged as an innovative platform for drug delivery across a wide range of human diseases, including myocardial infarction [5,6], stroke [7,8], infection and inflammation [9,10,11], hereditary disorders [12], cancer [13], and neurological conditions [14,15]. As natural carriers, exosomes possess unique properties: they are completely biocompatible, capable of penetrating tissues, and can be programmed for precise tissue targeting and drug loading—a significant departure from conventional nanomaterials like silver or gold particles, polymers, and liposomes [16,17]. The registration of over 260 clinical trials on ClinicalTrials.org as of March 2024 underscores the prominence of exosome-focused therapies in nanomedicine [4,18].

However, the transition of exosomes to clinical applications faces formidable challenges, particularly in quality assurance [19,20]. Synthesized and released from endocytic pathways, exosomes exhibit variability in composition, size, and cargo, making it challenging to standardize production processes and maintain consistent quality [21,22]. This variability is further compounded by the diversity of source cells as well as the genetic manipulations required for tissue-specific targeting or therapeutic loading [23]. Despite the widespread use of techniques such as reverse transcription-coupled polymerase chain reaction (RT-PCR), Western blot analysis, enzyme-linked immunosorbent assay, antibody array technology [24], and chips-based assays [25] in exosome research, these methodologies often prove laborious and low-throughput, failing to meet the demands for efficiently analyzing RNA and protein cargos simultaneously and effectively [26].

To address these challenges, we explore the potential of dual-omics—integrating transcriptomics and proteomics—as a comprehensive quality control measure for genetically engineered exosomes. Leveraging a genetic platform for surface modification of exosomes in human 293T cells, we utilize both endogenous and exogenous exosome-targeting scaffolds to create two sets of engineered exosomes [27,28]. Dual-omics analysis of these genetically modified exosomes as well as non-modified controls not only detected specific changes induced by genetic engineering, but also generated a profile of endogenous cargos and identified potential contaminants. Furthermore, pathway analysis conducted on key functional cargos such as miRNA and proteins showed a significant enrichment in a small subset of cellular signaling pathways, aiding in our understanding of the potential biological impacts that these exosomes may have on recipient cells. Our findings highlight the invaluable role of high-throughput methods in conducting thorough quality control assessments for genetically engineered exosomes.

## 2. Materials and Methods

### 2.1. Materials and Reagents

High glucose Dulbecco’s modified Eagle’s medium (DMEM) and OptiMEM were purchased from Gibco (Billings, MT, USA). UltraCULTURE serum-free medium, now discontinued, was purchased from Lonza (Hayward, CA, USA). Fetal bovine serum (FBS) was purchased from HyClone Laboratories (Logan, UT, USA). Penicillin–streptomycin and penicillin–streptomycin + L-Glutamine were purchased from Gibco (Billings, MT, USA). Polyethylenimine (PEI, Product No. 18978) was purchased from Millipore Sigma (St. Louis, MO, USA). ExoQuick-TC was purchased from System Biosciences (SBI, Palo Alto, CA, USA). Lentivectors expressing either neuron-targeting NCAM (XStamp™-NCAM) or exosome marker (XPack™) were purchased from System Biosciences (Palo Alto, CA, USA).

### 2.2. Expression Vectors for Genetic Engineering of Exosomes

Construction and validation of vectors for an exosome surface display system using either endogenous human tetraspanins (CD9-GFP, CD63-GFP, CD81-GFP) or exogenous vesicular stomatitis viral glycoprotein (VSVG-GFP) were previously reported [27,28]. Human CD47, CD47-Ecto, Transferrin fusion gene sequences were synthesized and subcloned into pcDNA3.1-C-GFP vectors by a service from Genscript (Piscataway, NJ, USA), resulting in CD47-GFP, CD47-Ecto-tVSVG-GFP, and hTransferrin-tVSVG-GFP expression vectors. Similarly, the murine truncated NCAM sequences from the XStamp^TM^-NCAM lentivector were used to assemble mNCAM-tVSVG-GFP and subsequently cloned in the lentivector CDH510B-1 from System Biosciences.

### 2.3. Human Cell Culture and Transfection

Human kidney 293T cells were cultured in DMEM medium supplemented with 10% FBS and 1% penicillin–streptomycin. Opti-MEM preparation contained 1% penicillin–streptomycin. UltraCULTURE preparations contained 1% penicillin–streptomycin + L-Glutamine. Cell transfections were conducted in 35 mm, 4-chamber glass-bottom plates. Plasmid DNA (1 µg/µL) and transfection reagent polyethylenimine (1 µg/µL) were prepared in Opti-MEM at a 1:5:100 ratio, incubated for 20 min, and introduced to the cell culture medium.

### 2.4. Molecular Tracking and Confocal Microscopy

Cultured 293T cells were imaged and captured using a Leica TCS SP8 confocal system. Each fluorescent and transmitted light image was captured using the same field at the 24, 48, and 72 h (h) post-transfection marks to monitor protein localization. All transfection and confocal imaging experiments were repeated at least once to ensure consistent results. Image adjustments (such as zoom, brightness, and contrast) were consistent across samples and applied to the whole frame of the image using Leica Application Suite X version 3.5.7.23225 software. Each selected image is representative of the whole cell population.

### 2.5. Exosome Preparation

Exosomes were isolated through a combination of centrifugation, ultrafiltration, and precipitation [12,29]. Briefly, 293T cells were transfected with DNA plasmid and cultured for 24 h, after which the medium was swapped to UltraCULTRE serum-free or Opti-MEM reduced serum medium. Opti-MEM was used for proteomics experiments due to discontinuation of UltraCULTRE by the manufacturer. After culturing for additional 48 h, the conditioned medium was collected, centrifuged at 1500× *g* for 10 min, and filtered through a 0.22 µm syringe filter. The medium was then mixed with ExoQuick-TC at a concentration of 1 mL ExoQuick-TC per 4 mL culture medium and incubated at 4 °C overnight. The solution was then centrifuged at 3000× *g* for 90 min, and the supernatant was discarded. The resulting pellet was re-suspended in phosphate buffer solution (PBS) and stored at −20 °C for further study.

### 2.6. Nanoparticle Tracking Analysis (NTA)

NTA analysis was performed in an aqueous environment, as reported previously [9]. Exosome samples were diluted 20-fold in PBS for NTA analysis. The size distribution and concentration of isolated exosomes were measured using a NanoSight LM10 instrument with 405 nm and 60 mV laser sources. The data were analyzed using NTA software (NTA version 2.3 build 0033, Malvern Instruments Ltd., Malvern, UK), which included size distributions that were calculated and graphed. NTA analysis was carried out using a service provided by Particle Characterization Laboratories, Inc. (Novato, CA, USA).

### 2.7. Slot Blot Analysis

The investigation of various exosome markers was conducted through a slot blot immunological analysis employing pre-manufactured antibody arrays from SBI (Palo Alto, CA, USA). Each blot, containing 12 pre-printed slots, featured 8 antibodies targeting exosome markers (CD63, CD81, ALIX, FLOT1, ICAM1, EpCAM, ANXA5, TSG101) along with cytosolic GM130. About 50 μg of exosome proteins were employed in each assay, adhering to the immuno-binding and detection protocol outlined in the provided user manual. The image was recorded on Image Lab version 6.1 using a Bio-Rad Gel Doc XR+ Imaging system with an exposure time of 1.0 s.

### 2.8. Next-Generation Sequencing (NGS)

Exosomes isolated from conditioned medium from both the control and differently engineered samples were processed for NGS analysis using SBI’s transcriptomics services [30,31]. The Illumina NextSeq platform was used for NGS, and the identification of nucleobase quality was performed using Phred quality scoring under Sanger/Illumina 1.9 encoding. RNA data was then mapped to the GRCh38 human reference genome. Data were returned from SBI in the form of raw FastQ files as well as Excel files containing counts, RNA id, RNA type, and chromosomal locations for each mapped RNA.

### 2.9. Liquid Chromatography–Mass Spectrometry (LC-MS)

Exosomes isolated from conditioned medium from both control and differently engineered samples were processed for LC-MS analysis by SBI’s proteomics service [32]. Briefly, 10 μg of exosome protein from each sample was subjected to electrophoresis using sodium dodecyl-sulfate polyacrylamide gel electrophoresis (SDS-PAGE) and in-gel digestion with trypsin using a ProGest robot (DigiLab, Hopkinton, MA, USA). Half of the digested sample was processed by the nano LC-MS/MS with a Waters NanoAcquity Ultra-Performance Liquid Chromatography (UPLC) system interfaced to a ThermoFisher Q Exactive. Peptides were loaded on a trapping column and subsequently eluted using a reverse-phase gradient. The mass spectrometer was operated in a data-dependent mode and the Orbitrap operated at 60,000 full width at half maximum (FWHM) and 17,500 FWHM for MS and tandem mass spectrometry (MS-MS), respectively. The fifteen most abundant ions were selected for MS-MS.

### 2.10. Dual-Omics Analysis and Protein Contaminant Detection

Nonhuman RNA mapping for the detection of VSVG and GFP sequences was performed using Geneious Prime 2023.0.4. All Truseq, Nextera, and PhiX 3’ adapters were trimmed using the BBDuk data-quality trimming, filtering, and masking plugin. The adapters were trimmed using a k-mer length of 27 and a maximum of 1 substitution. Low-quality reads were trimmed at both the 5′ and 3′ ends, and reads shorter than 10 base pairs were discarded. NGS and LC-MS data were analyzed using Matlab and Python scripts, as well as Excel functions. Transcript counts was trimmed at count ≥25 before analysis. Statistical analysis was performed using Python scripts and Excel functions. LC-MS contaminant detection was performed via Python scripts and the use of the Uniprot and Ensembl protein databases.

### 2.11. Pathway Analysis

Pathway analysis was performed via FunRich version 3.1.4 [33] using their built-in database and the 12 October 2023 release of Vesiclepedia [34]. Pathway analysis was performed for both miRNAs and proteins by comparing expression levels against 131 and 52 cellular signal/metabolic pathways, respectively. All bovine contaminant proteins were excluded from this pathway analysis. Species that could not be mapped to a known biological pathway, as well as any pathway containing less than 5% of total biological activity, were removed, and the rest of the pathways were tabulated. *p*-values were generated for each pathway, and *p* values > 0.05 represent significant enrichment of a selected pathway compared to the background data set (FunRich Database).

## 3. Results

### 3.1. System Design and Genetic Engineering of Exosomes in Human 293T Cells

**Figure 1:**
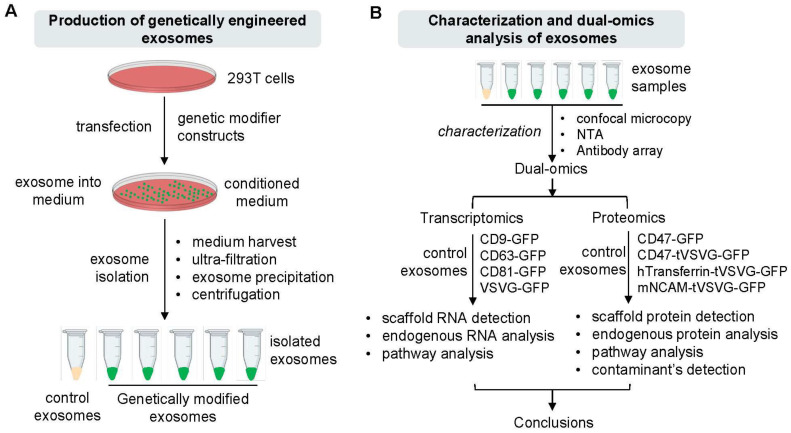
Production and characterization of genetically modified exosomes in cultured human 293T cells. Schematic representation of the production and isolation of exosomes (**A**), which are used for further characterization and dual-omics analysis (**B**). After initial characterization studies, engineered exosomes were divided into two cohorts, which were subjected to next-generation sequencing analysis of whole transcripts (transcriptomics) or liquid chromatography coupled with mass spectrometry analysis (LC-MS, proteomics).

**Figure 2:**
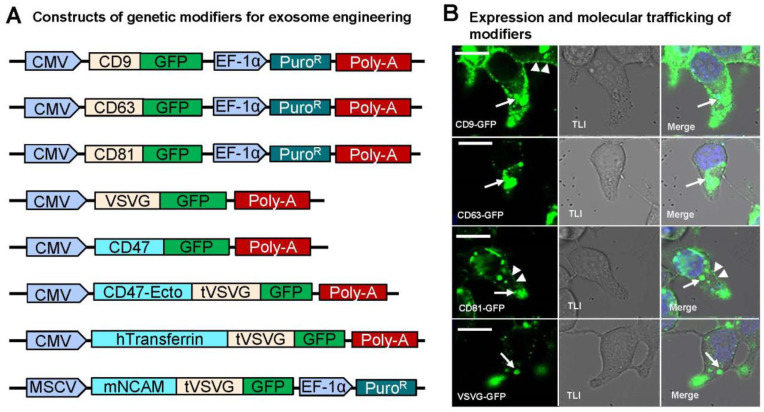
Construct schematics and scaffold expression. (**A**) Schematic representation of constructs containing exosome-targeting scaffolds (CD9, CD63, CD81, VSVG) and molecular modifiers (GFP, CD47, mNCAM, Transferrin). (**B**) Confocal image study confirms successful scaffold targeting to sites of exosomal biogenesis (the plasma membrane and endocytic compartments). 293T cells were transfected with fusion protein constructs including CD9-GFP, CD63-GFP, CD81-GFP, and VSVG-GFP. Fluorescence images were recorded 72 h after transfection. The transmitted light images (TLI) show the corresponding morphology of the imaged cells. The punctuated plasm membrane (indicated by arrowheads) and endocytic compartment (indicated by arrows) became apparent when the fluorescent images were merged with the blue nuclei stained with HOCHEST. Scale bar, 10 μm.

To enable targeted exosome-based therapeutics with enhanced specificity and payload delivery, we designed and implemented two sets of genetic constructs for modifying exosomes in cultured human 293T cells (Figure 1A,B). The first set was composed of endogenous exosome-targeting scaffolds (CD9, CD63, CD81) and exogenous viral envelope proteins like vesicular stomatitis viral glycoprotein (VSVG), each fused with a green fluorescence protein (GFP) at the C-terminus for live cell monitoring and detection (Figure 2A). The second set included genetic modifiers such as CD47, human transferrin (hTransferrin), and mouse neural cell adhesion molecule (mNCAM) sequences, aimed at functionalizing exosomes for specific purposes. Specifically, CD47 possesses a potent anti-phagocytosis signal domain, therefore potentially increasing the half-life of exosomes in circulation [35,36]. Transferrin may enhance blood-brain barrier (BBB) crossing via receptor-mediated epithelial transcytosis [37]. NCAM may enhance neuron targeting via molecular interactions [38]. These modifiers, lacking intrinsic exosome-targeting signal peptides, were fused with VSVG for targeting. These constructs also included the same GFP monitoring tag as the first set of genetic constructs. The amino acid sequences and coding DNAs of the second sets of genetic modifiers are provided in the Supplementary Materials Sequence File. All constructs were tagged with GFP for molecular tracking and imaging. To ensure all constructs were properly expressed and targeted to exosome biogenic sites, fluorescence images of cultured 293T cells were recorded following transfection. Fluorescence imaging of transfected 293T cells confirmed the proper expression and targeting of all constructs to exosome biogenic sites. GFP fusion proteins (CD9-GFP, CD63-GFP, CD81-GFP, VSVG-GFP) appeared on either the plasma membrane (Figure 2B, arrowheads) or punctate in the cytosol (Figure 2B, white arrows), consistent with lipid raft or endocytic localization. Similarly, CD47-GFP, CD47-Ecto-VSVG-TM-GFP, hTransferrin-VSVG-TM-GFP, and mNCAM-VSVG-TM-GFP showed exosome-associated expression and subcellular localization (Figures S1 and S2). Co-transfection experiments with known exosome markers (CD63 or XPACK) confirmed the colocalization of GFP-tagged exosome modifiers with RFP-tagged exosome markers, indicating integration into exosomes (Figures S1 and S2). We typically monitor and record confocal images at different time points, such as 24, 48, and 72 h. Due to the similarity in imaging patterns across these time points, we chose to present images from one time point to be representative.

Taken together, these results strongly support the functionality of our genetic modifiers, suggesting their integration into exosomes via endogenous biogenic pathways under our experimental conditions.

### 3.2. Preparation and Characterization of Genetically Engineered Exosomes

To assess the maturation and release of genetically modified exosomes into the cell culture medium, we isolated exosomes from conditioned media using established protocols [25]. These protocols used a combination of ultrafiltration (pore size ≤ 200 nm), chemical precipitation, and low-speed centrifugation to ensure the isolation of quality exosomes (size ≤ 200 nm) rather than microvesicles (≥200 nm). To enhance the purity and to minimize potential contaminants for bovine serum, OptiMEM or UltraCulture medium without serum-supplement was used for all exosome harvests.

**Figure 3:**
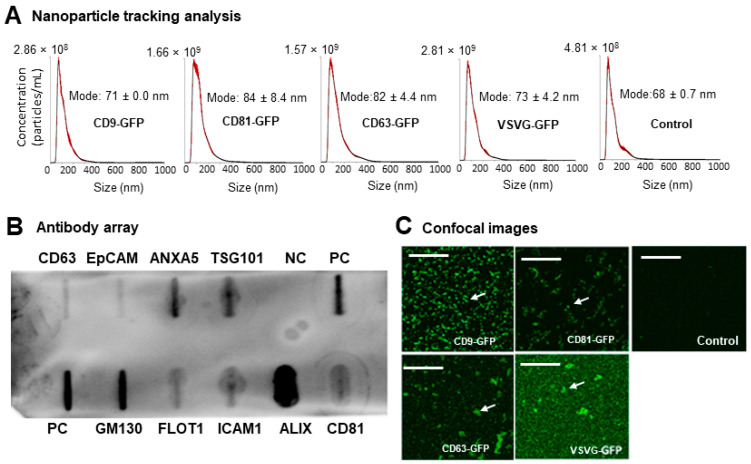
Characterization of genetically modified exosomes and non-modified controls. (**A**) Exosome size and distribution determined via nanoparticle tracking analysis (NTA). NTA profiles of CD9–GFP-, CD63–GFP-, CD81–GFP-, and VSVG–GFP-modified exosomes isolated from 293T cells at Day 3 post-transfection, showing the particle size and distribution of the respective engineered exosomes and the non-engineered control. (**B**) Exosome marker analysis via antibody array. The immuno-slot assay was able to detect different levels of cellular proteins loaded onto exosomes. PC: positive control; NC: negative control. (**C**) Fluorescence confocal images of isolated, genetically engineered exosomes (CD9–GFP, CD63–GFP, CD81–GFP, and VSVG–GFP) and non-engineered controls. White arrows indicate GFP-positivity for engineered exosomes, where background GFP noise is shown in the control sample. Scale bar, 50 μm.

Following the preparation of genetically engineered exosomes, we conducted various analyses to determine their size distribution, the presence of major exosome markers, and proper modification by our genetic constructs. As depicted in Figure 3A and Figure S3, all exosome preparations exhibited similar particle size distributions compared to unmodified controls, with a single peak in the size range of 58~85 nm, consistent with the exosome size range (<150 nm). Additionally, immune slot blot analysis using an antibody array revealed different levels of expression for all eight exosome markers (CD63, CD81, ALIX, FLOT1, ICAM1, EpCAM, ANXA5, and TSG101), with no bands observed in negative controls (Figure 3B), confirming the predominance of exosomes in our preparations. To further qualitatively assess the proper modification of prepared exosomes by our genetic scaffolds or modifiers, confocal imaging was conducted on both genetically modified exosomes and non-modified controls. As depicted in Figure 3C, confocal images showed strong GFP signals in genetically modified exosomes compared to non-modified controls, indicating successful targeting and loading of GFP-containing genetic modifiers onto exosomes.

In summary, these results demonstrate that these constructs effectively produced genetically modified exosomes with a size distribution and characteristics that match the exosome subtype.

### 3.3. Dual-Omics Identifies Engineering-Induced Changes

**Figure 4:**
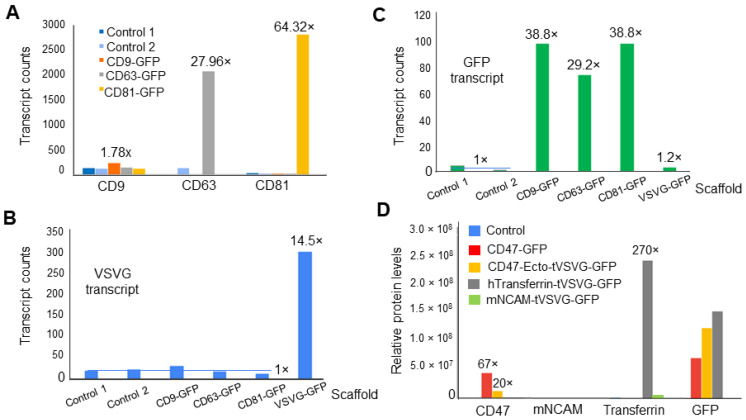
NGS sequencing unveiled enhanced levels of engineering scaffolds and GFP as compared to controls. (**A**) Human genome mapping of NGS data reveals elevated levels of scaffold proteins in their associated samples, including CD9 (1.78×), CD63 (27.96×), and CD81 (64.32×). Additional analysis via Geneious Prime highlights elevated levels of VSVG (14.5×) in the VSVG-GFP transfected sample (**B**) and GFP (29.2×–38.8×) in CD9, CD63, and CD81 samples (**C**). LC-MS detected increased levels of expression for CD47 in CD47-GFP (67×) and CD47-tVSVG-GFP (20×), human Transferrin in hTransferrin-tVSVG-GFP (270×), and GFP in GFP-modified exosome samples (except mNCAM-tVSVG-GFP), but not in control exosomes. LC-MS was not able to detect murine NCAM proteins (**D**).

To assess the capability of dual-omics to discern specific changes induced by engineering constructs, we isolated exosomes from both genetically modified producer cells and non-modified controls. The total RNA extracted from these exosomes underwent NGS and transcriptomic analysis. The engineering groups consistently exhibited increased levels of mRNA transcripts associated with their engineering scaffolds (CD9, CD63, CD81, VSVG) and the fluorescent marker (GFP), as depicted in Figure 4A,B. These scaffold transcripts (CD9, CD63, CD81) were successfully detected through mapping to the GRCh38 human reference genome (Figure 4A) or Geneious Prime mapping software version 2023.0.4 for viral VSVG (Figure 4B). Notably, the CD63-GFP, CD81-GFP, and VSVG-GFP samples showed significant increases in associated mRNAs (27.96×, 64.32×, and 14.5×, respectively), while the CD9 transcript exhibited only a slight elevation (1.78×), influenced by detection efficacy and base levels. Figure 4C illustrates that all engineered samples, except VSVG-GFP, demonstrated significant levels of the GFP fluorescence marker. It is important to note that the increases in the levels of different scaffold transcripts varied, a discrepancy which may have been caused by variations in the levels of endogenous gene expressions for CD9/CD63/CD81, batch-to-batch transfection efficiency, and/or sensitivity of NGS analysis. Nevertheless, these findings confirm that NGS effectively detected elevated levels for most of engineering scaffolds and reporter transcripts, indicating proper expression of genetic constructs in producer cells. In parallel, we employed LC-MS proteomics to evaluate specific changes induced by engineering constructs at the protein level. Exosomes from genetically modified and control samples underwent LC-MS analysis. The quantification of protein modifiers revealed increased levels of CD47 in both CD47 constructs (67× for CD47-GFP and 20× for CD47-Ecto-tVSVG-GFP) (Figure 4D). Similarly, human transferrin levels showed a significant 270-fold increase in the hTransferrin-tVSVG-GFP-engineered sample compared to other modifiers, as well as the non-modified control (Figure 4D). Consistently, LC-MS also detected GFP proteins in all engineered groups except mNCAM-tVSVG-GFP, but did not detect GFP in the non-modified controls. Notably, LC-MS failed to detect murine NCAM in mNCAM-tVSVG-GFP-modified exosomes, indicating that the detection of proteins may differ in sensitivity. Using confocal microscopy for the direct detection of protein integration into exosomes is crucial for visual confirmation (Figure 3C). However, omics analysis complements confocal microscopy by providing comprehensive insights into protein composition and functional attributes, enhancing the credibility and depth of our findings regarding engineered exosomes [39,40].

Taken together, our results strongly support that dual-omics can effectively identify specific changes induced by genetic engineering, confirming the successful modification of exosomes at both the transcript and protein levels.

### 3.4. Dual-Omics Identifies a Diverse Ranges of Endogenous Cargos

**Figure 5:**
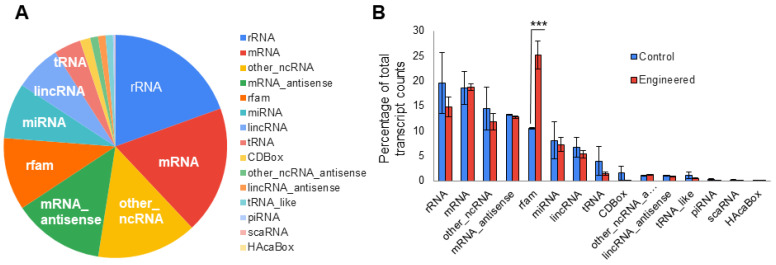
NGS sequencing identifies fifteen RNA types and reveals differences between engineered and control samples in key RNA subtypes. (**A**) Transcriptomics analysis reveals the presence of fifteen RNA types present in varying quantities in control samples (*n* = 2). (**B**) Comparison between control (*n* = 2) and engineered (*n* = 4) samples highlights significant differences in relative abundance of RNA transcripts in some subtypes (*** indicates *p* ≤ 0.001). Error bars show the standard error of the mean (SEM).

To comprehensively understand the transcript cargos in exosomes, we set a threshold of 25 counts or higher as positive in isolated exosome samples. The relative abundance of all RNA species in the two control samples was then averaged and is presented in Figure 5A. The results clearly demonstrate the presence of all fifteen types of RNAs in exosomes isolated from human 293T cells, with major RNA species including rRNA (19.4%), mRNA (18.5%), uncategorized non-coding RNAs or other_ncRNA (14.5%), rfam (10.5%), miRNA (8.1%), lincRNA (6.7%), and tRNA (3.9%). The remaining RNA species, such as CDBox, other noncoding RNAs, lincRNA antisense, tRNA_like molecule, piRNA, caRNA, and HAcaBox, collectively constituted less than 6%. To determine whether genetic modification of exosomes may alter the relative abundance of transcript cargos, we compared the abundance of various RNA species in engineered exosome samples (*n* = 4) vs. controls (*n* = 2). Figure 5B illustrates that only rfam RNAs exhibited significant increases in abundance in engineered exosomes compared to controls (25.2% vs. 10.5%, 2-tailed *t*-test), while all 14 remaining RNA species showed similar levels between sample arms. These findings suggest that genetic engineering may not substantially impact the loading of endogenous RNA cargos.

**Figure 6:**
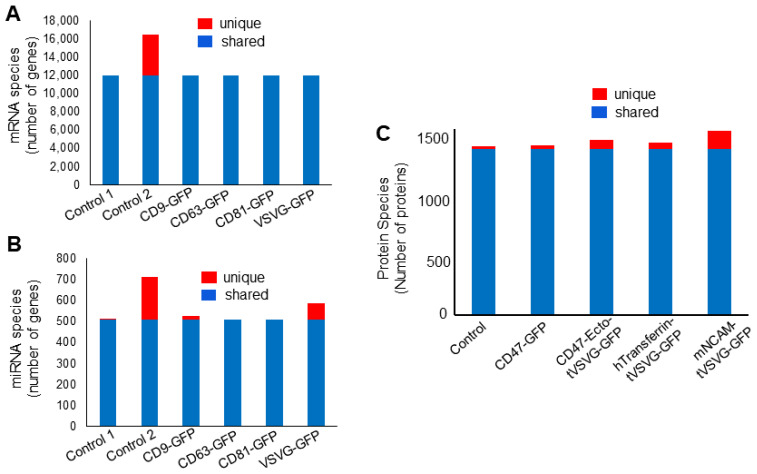
Global profile of transcript and protein cargos by dual-omics. (**A**) Cross-sample comparison of mRNAs (count ≥ 25) reveals a cohort of shared mRNAs (11,996) and minimal differences between control and engineered samples prepared under the same culture conditions (Control 1, CD9, CD63, CD81, VSVG). Control 2, prepared under different culture conditions, contains the most variation, with 4485 unique mRNAs. (**B**) Cross-sample comparison of miRNAs (count ≥ 25) highlights a group of shared miRNAs (511) and minor differences between Control 1 and CD9 (19) and VSVG (78) engineered samples. Control 2, prepared from different batches, retains the most variation, with 205 unique miRNAs. (**C**) Cross-sample comparison of LC-MS protein data shows shared proteins (*n* = 1338) in all samples and unique proteins (*n* = 23–148) in individual samples.

To gain further insight into the potential impacts of genetic engineering on two biologically significant exosomal RNA species, mRNA and miRNA, we conducted a detailed comparison among genetically engineered exosome samples (*n* = 4) and non-engineered controls (*n* = 2) (Figure 6A,B). The results showed that most RNAs were shared among all samples, including 11,996 mRNAs and 507 miRNAs. Differences in unique RNA species were mainly between two controls (prepared from different batches) rather than amongst control 1 and the engineered samples. These results support that genetic engineering processes may not substantially alter RNA cargo loading, particularly for the two biologically significant types of RNAs (mRNA and miRNA). Consistent with our findings for RNA cargos, LC-MS proteomics identified over 1656 unique proteins that were loaded and/or associated with exosomes. Among them, 1338 proteins (80.8%) were shared among samples, while a smaller grouping of proteins (23~148) was unique to each sample, totaling 318 proteins or 18.2% (Figure 6C). It appears that there were more unique proteins in engineered samples (28~148) than in the control (23), suggesting a potential impact on protein cargo loading by our genetic engineering processes.

### 3.5. Impact on Cellular Signaling and Metabolism by Endogenous Exosome Cargos

To investigate the potential biological effects of exosome cargos on cellular processes such as signaling and metabolism, we used the FunRich functional tool to examine the enrichment of biologically active cargos in both genetically engineered exosomes and the controls. Our analysis focused on two important classes of biologically active cargos, namely, miRNAs and proteins, which regulate 131 and 52 defined biological pathways, respectively (Tables S1 and S2). The results revealed significant enrichment of exosome miRNAs in only a small subset of cellular pathways (4 out of 131), including miRNA species involved in regulating signal transduction, cell communication, nucleic acid metabolism, and transport (Table 1). Importantly, comparisons between control and engineered exosome samples demonstrated minimal dissonance in pathway abundance, with a difference of ≤1% for the pathways listed in Table 2. This implies that genetic engineering may not significantly alter exosomal miRNA cargo loading, or, consequently, the relative abundance of individual miRNA species. Next, the FunRich program was applied to proteomics data using the UniProt protein database. Remarkably, we found that protein cargos were significantly enriched in a small subset of cellular pathways (4 out of 52) in both engineered exosomes and controls. These pathways encompassed signal transduction, cell communication, nucleic acid metabolism, and overall metabolism (Table 2).

In summary, our pathway analysis indicates that the enrichment of exosome cargos may occur in a small subset of cellular pathways for both miRNA (4 out of 131; ~3%) and proteins (4 out of 52; ~7.7%), suggesting a selective loading of bioactive cargos in 293T cells. Moreover, these enrichments tend to occur in highly regulated pathways, implying potential biological impacts on cellular biology and/or pathology of recipient cells via these exosome cargos.

### 3.6. Proteomics Is Able to Identify Potential Protein Contaminants

LC-MS proteomics, known for its high-throughput capability, is adept at detecting proteins from diverse sources, making it well-suited for a thorough contaminant analysis. To evaluate its effectiveness in genetically engineered exosomes, we conducted a comparative analysis on genetically modified exosomes and their non-modified controls. The results demonstrate that the LC-MS method can identify various protein contaminants, including 99 cow proteins (Table 3). These proteins comprise 93 proteins from cow plasma and 6 proteins without known tissue origin, likely stemming from the use of fetal bovine serum in the culture media. Additionally, another two contaminants, trypsin and streptavidin, were also detected (Table 3). The presence of trypsin in our isolated exosomes is likely attributed to its use in the cell culture protocol, particularly during trypsinization of the cells and ingel digesting of exosome proteins during the LC-MS processes. Furthermore, the identification of streptavidin aligns with its role in the LC-MS processes.

## 4. Discussion

Ensuring the quality control of exosome-based therapeutics is a challenging task due to the heterogeneous nature of exosomes and the complexities involved in the engineering processes. Addressing these challenges requires a high-throughput approach to quality control, recognizing the complexity of exosomes and the potential impact of exosome variations on therapeutic efficacy and safety. Developing robust quality control measures is crucial in order to capture and characterize the variability of exosome populations as well as specific modifications introduced through genetic engineering, facilitating the smooth translation of exosome-based therapeutics into clinical applications.

Using two cohorts of genetically engineered exosomes as examples, we demonstrated the effectiveness of a dual-omics approach in enhancing the quality control of engineered exosomes. This approach generated a substantial amount of high-quality data, showcasing its effectiveness. For instance, the NGS produced approximately 15 million reads/sample, with over 80% mapping to the human genome. These sequences encompassed 15 different RNA species, including 13,897 mRNAs and 2008 miRNAs (Figure 6A,B). Similarly, LC-MS methods generated thousands of unique peptides, identifying over 1656 unique human proteins that were loaded onto exosomes (Figure 6C). Additionally, the LC-MS method was able to detect nonhuman proteins, including 99 cow proteins, highlighting its efficacy in detecting potential contaminants and their sources (Table 3). Furthermore, pathway analysis of exosome cargos revealed selective enrichment of both miRNAs and proteins in regulating cellular signaling and metabolism, providing important insights into biological impacts on recipient cells (Table 1 and Table 2). For example, miRNA cargos were more enriched in regulating cell communication and signaling pathways, while protein cargos were more involved in cellular metabolism. These results indicate that these important bioactive cargos may influence different biological activities via distinct pathways. The high-throughput capability and quality of sequence data obtained through this dual-omics approach are clearly more advantageous to the characterization of genetically modified EVs than conventional methods such as RT-PCR, Western blot, and ELISA. Exosomes from different cell types can exhibit distinct characteristics due to their cellular origins. Studies have shown that the protein and RNA content of exosomes can vary significantly depending on the source cell type, influencing their biological functions and therapeutic potential [41,42]. Therefore, while our current study focuses on 293T cells, future research will explore the properties of exosomes derived from other cell types to fully understand their potential and applications.

We utilized different plasmids in our dual-omics study to explore the impact of various genetic modifications on exosome quality. Each plasmid targeted distinct engineering scaffolds, offering a broader perspective on the influence of different genetic constructs on exosome characteristics. This strategy ensured the collection of more comprehensive and diverse data [26]. While the dual-omics approach yields a substantial amount of high-quality data regarding transcripts and protein cargos, it is important to note that each dataset may have limitations and potential biases toward specific cargos. For instance, NGS identified a higher number (*n* = 13,897) of mRNAs, while LC-MS detected a smaller number of protein products (*n* = 1656), indicating the different sensitivities of the two methods. In some cases, sequence information may only be obtained from one dataset, but not the other. For instance, certain transcript cargos, such as noncoding RNAs (rRNA, miRNA, lincRNA), may exclusively appear in NGS data, while protein contaminants are typically identified by the LC-MS method. Furthermore, NGS was unable to identify non-human transcripts such as VSVG and GFP without additional data processing, and LC-MS was unable to detect certain proteins such as truncated VSVG and murine NCAM, indicating some limitations regarding their use with nonhuman transcripts or proteins. Additionally, the lack of detectable GFP transcripts and/or proteins in cells transfected with VSVG-GFP or mNCAM-tVSVG-GFP (Figure 4C,D) may have been due to differences in fusion sequences, the ability of exosomes to incorporate the transcript or protein, and the sensitivity of the detection methods used for various transcripts or proteins within exosomes [43]. The dual-omics analysis reveals differences in RNA transcripts and protein expression in engineered exosomes, providing crucial information about their molecular composition and functionality. This comprehensive analysis ensures that the engineered exosomes meet desired specifications and perform as intended, helping to optimize both transcriptomic and proteomic protocols for the accurate and reliable detection of target molecules [4,44]. Nevertheless, these experiments provide strong support for the conclusion that dual-omics may complement each other, providing a more comprehensive picture of exosome contents than individual omics.

Although our dual-omics approach effectively addresses two biologically important cargos, namely, transcripts and proteins, it is crucial to acknowledge that other biological cargos such as lipids and metabolites may require additional analytical methods. The question of whether lipidomics can contribute additional value to the quality control process remains unanswered. Further exploration is needed to determine the applicability and benefits of integrating lipidomics into the dual-omics approach for a more complete understanding of exosome composition and quality. Nevertheless, dual-omics analysis aids in quality control of engineered exosomes by offering comprehensive insights into both RNA transcripts and protein expression profiles. By comparing these profiles between engineered and control exosomes, we can assess the efficacy of genetic modifications and ensure consistency in exosome composition. This approach enables a holistic evaluation of exosome quality, facilitating the optimization of engineered exosome-based therapeutics.

In summary, this dual-omics-based approach can aid in quality control of genetically engineered exosomes via identification of numerous endogenous cargos and exogenous engineering modifiers, as well as potential protein contaminants. Pathway analysis may predict potential biological impacts on recipient cells by endogenous cargos. This study presents a new framework for the use of the high-throughput technologies, particularly transcriptomics and proteomics, in the pursuit of comprehensive quality controls of exosome-based therapeutics.

## Figures and Tables

**Table 1:** Percentage of miRNAs involved in regulating various cellular pathways.

Pathway	Control 1	Control 2	CD9	CD63	CD81	VSVG
Cell Growth and Maintenance	6.2	6.1	6.2	6	6	6.2
Protein Metabolism	7	6.9	7	7	7	6.9
Energy Pathways	7.4	7.5	7.4	7.4	7.4	7.7
Transport	7.6 ***	7.7 ***	7.6 ***	7.5 ***	7.5 ***	7.7 ***
Metabolism	7.7	8	7.7	7.7	7.7	7.8
Nucleic Acid Metabolism	17.6 ***	17.5 ***	17.6 ***	17.7 ***	17.8 ***	17.7 ***
Cell Communication	21.7 ***	21.2 ***	21.6 ***	21.8 ***	21.9 ***	21.4 ***
Signal Transduction	23.3 ***	22.8 ***	23.2 ***	23.3 ***	23.4 ***	23.1 ***

Note: miRNA isolated from non-modified exosomes or modified exosomes using CD9, CD63, CD81, or VSVG scaffolds. *** *p* < 0.001 indicates significant enrichment above background data set (FunRich Database).

**Table 2:** Percentage of proteins involved in regulating various cellular pathways.

Pathway	Control	CD47-GFP	CD47-tVSVG-GFP	hTransferrin-tVSVG-GFP	mNCAM-tVSVG-GFP
Transport	8.4	8.3	8.2 ***	8.1	8.8
Cell Growth and Maintenance	11.7 ***	10.4 ***	10.3 ***	10.3 ***	9.9 ***
Energy Pathways	13.2 ***	13.5 ***	13.8 ***	13.5 ***	13 ***
Metabolism	13.5 ***	13.8 ***	14 ***	13.7 ***	13.1 ***
Nucleic Acid Metabolism	16.8	17.1	17.1	17.6	17.8
Cell Communication	17.5	17.1	16.4	16.6	16.5
Protein Metabolism	18.3 ***	18.2 ***	17.9 ***	18.1 ***	17.8 ***
Signal Transduction	18.4	18.4	17.6	17.8	17.9

*** *p* < 0.001, indicates significant enrichment above background data set (Uniprot Database).

**Table 3:** Contaminants of exosome preparation detected by LC-MS analysis.

Sample	Cow Plasma Proteins	Unknown Cow Proteins	Trypsin	Streptavidin
Control	92	6	Yes	Yes
CD47-GFP	91	6	Yes	Yes
CD47-Ecto-tVSVG	93	6	Yes	Yes
hTransferrin-tVSVG-GFP	91	6	Yes	Yes
mNCAM-tVSVG-GFP	91	6	Yes	Yes

## Data Availability

All data generated or analyzed in this study is included in the published article and its Supplementary sequence files. Data and experimental materials are available from the corresponding author upon reasonable request.

## Supplementary Materials

The following supporting information can be downloaded at: https://www.mdpi.com/article/10.3390/pharmaceutics16060824/s1, Figure S1: Expression and molecular trafficking of genetic modifiers of exosomes in living human 293T cells; Figure S2: Expression and co-localization of genetic modifiers with exosome markers of CD63 and XPACK in cultured human 293T cells; Figure S3: Nanoparticle tracking analysis of isolated exosomes with various genetic modifications (A–D) or non-modification (E). Table S1: 131 Cellular Pathways Regulated by miRNAs; Table S2: 52 Cellular Pathways Regulated by Proteins; Table S3: Summary of 99 Protein Contaminants and Their Relative Abundance in Control and Engineered Exosomes; Supplementary sequence file including fusion proteins and their coding sequences for constructs.
