# Supplementary material for: Dual-Omics Approach Unveils Novel Perspective on the Quality Control of Genetically Engineered Exosomes
Source: Pharmaceutics. 2024 Jun 18;16(6):824. doi: 10.3390/pharmaceutics16060824 (PMC11207238; doi:10.3390/pharmaceutics16060824)
Supplement: Supplementary file 1 [file pharmaceutics-16-00824-s001.zip › Sequence File.pdf]

Supplementary sequence file

The following document includes fusion proteins and their coding sequences for constructs: (1) CD47-GFP; (2) CD47-Ecto-tVSVG-GFP; (3) hTransferrin-tVSVG-GFP; and (4) mNCAM-tVSVG-GFP.

1. **CD47-GFP**

Encoded protein (580 aa)

MWPLVAALLLGSACCGSAQLLFNKTKSVEFTFCNDTVVIPCFVTNMEAQNTTEVYVKWKFKGRDIYTFDGALNKS
TVPTDFSSAKIEVSQLLKGDASLKMDKSDAVSHTGNYTCEVTELTREGETIIELKYRVVSWFSPNENILIVIFPIFAILLF
WGQFGIKTLKYRSGGMDEKTIALLVAGLVITVIVIVGAILFVPGEYSLKNATGLGLIVTSTGILILLHYYVFSTAIGLTSFV
IAILVIQVIAYILAVVGLSLCIAACIPMHGPLLISGLSILALAQLLGLVYMKFVASNQKTIQPPRKAVEEPLNAFKESKG
MMNDEGSTSPVWWNSADIQHSGGRMSKGEELFTGVVPILVELDGDVNGHKFSVSGEGEGDATYGKLTLKFICTT
GKLPVPWPTLVTTLTYGVQCFSRYPDHMKQHDFFKSAMPEGYVQERTIFFKDDGNYKTRAEVKFEGDTLVNRIEL
KGIDFKEDGNILGHKLEYNYNSHNVYIMADKQKNGIKVNFKIRHNIEDGSVQLADHYQQNTPIGDGPVLLPDNHYL
STQSALSKDPNEKRDHMVLLEFVTAAGITHGMDELYK*

Annotation: human CD47 and eGFP

Coding sequences of CD47-GFP

atgTGGCCCCTGGTAGCGGCGCTGTTGCTGGGCTCGGCGTGCTGCGGATCAGCTCAGCTACTATTTAATAAAA
CAAAATCTGTAGAATTCACGTTTTGTAATGACACTGTCGTCATTCCATGCTTTGTTACTAATATGGAGGCACAA
AACACTACTGAAGTATACGTAAAGTGGAAATTTAAAGGAAGAGATATTTACACCTTTGATGGAGCTCTAAACA
AGTCCACTGTCCCCACTGACTTTAGTAGTGCAAAAATTGAAGTCTCACAATTACTAAAAGGAGATGCCTCTTTG
AAGATGGATAAGAGTGATGCTGTCTCACACACAGGAAACTACACTTGTGAAGTAACAGAATTAACCAGAGAA
GGTGAAACGATCATCGAGCTAAAATATCGTGTTGTTTCATGGTTTTCTCCAAATGAAAATATTCTTATTGTTATT
TTCCCAATTTTTGCTATACTCCTGTTCTGGGGACAGTTTGGTATTAAAACACTTAAATATAGATCCGGTGGTAT
GGATGAGAAAACAATTGCTTTACTTGTTGCTGGACTAGTGATCACTGTCATTGTCATTGTTGGAGCCATTCTTT
TCGTCCCAGGTGAATATTCATTAAAGAATGCTACTGGCCTTGGTTTAATTGTGACTTCTACAGGGATATTAATA
TTACTTCACTACTATGTGTTTAGTACAGCGATTGGATTAACCTCCTTCGTCATTGCCATATTGGTTATTCAGGTG
ATAGCCTATATCCTCGCTGTGGTTGGACTGAGTCTCTGTATTGCGGCGTGTATACCAATGCATGGCCCTCTTCT
GATTTCAGGTTTGAGTATCTTAGCTCTAGCACAATTACTTGGACTAGTTTATATGAAATTTGTGGCTTCCAATC
AGAAGACTATACAACCTCCTAGGAAAGCTGTAGAGGAACCCCTTAATGCATTCAAAGAATCAAAAGGAATGA
TGAATGATGAAGGATCCACTAGTCCAGTGTGGTGGAATTCTGCAGATATCCAGCACAGTGGCGGCCGCATGA
GCAAGGGCGAGGAGCTGTTCACCGGGGTGGTGCCCATCCTGGTCGAGCTGGACGGCGACGTAAACGGCCAC
AAGTTCAGCGTGTCCGGCGAGGGCGAGGGCGATGCCACCTACGGCAAGCTGACCCTGAAGTTCATCTGCACC
ACCGGCAAGCTGCCCGTGCCCTGGCCCACCCTCGTGACCACCCTGACCTACGGCGTGCAGTGCTTCAGCCGCT
ACCCCGACCACATGAAGCAGCACGACTTCTTCAAGTCCGCCATGCCCGAAGGCTACGTCCAGGAGCGCACCAT
CTTCTTCAAGGACGACGGCAACTACAAGACCCGCGCCGAGGTGAAGTTCGAGGGCGACACCCTGGTGAACCG
CATCGAGCTGAAGGGCATCGACTTCAAGGAGGACGGCAACATCCTGGGGCACAAGCTGGAGTACAACTACA
ACAGCCACAACGTCTATATCATGGCCGACAAGCAGAAGAACGGCATCAAGGTGAACTTCAAGATCCGCCACA
ACATCGAGGACGGCAGCGTGCAGCTCGCCGACCACTACCAGCAGAACACCCCCATCGGCGACGGCCCCGTGC
TGCTGCCCGACAACCACTACCTGAGCACCCAGTCCGCCCTGAGCAAAGACCCCAACGAGAAGCGCGATCACA
TGGTCCTGCTGGAGTTCGTGACCGCCGCCGGGATCACTCACGGCATGGACGAGCTGTACAAGTAA

2. CD47-Ecto-tVSVG-GFP

Coded protein (523aa)

MWPLVAALLLGSACCGSAQLLFNKTKSVEFTFCNDTVVIPCFVTNMEAQNTTEVYVKWKFKGRDIYTFDGALNKS
TVPTDFSSAKIEVSQLLKGDASLKMDKSDAVSHTGNYTCEVTELTREGETIIELKYRVVSWFSPNENTTTPAPRPPTP
APTIASQPLSLRPEACRPAAGGAVHTRGLDFACDEHPHIQDAASQLPDDESLFFGDTGLSKNPIELVEGWFSSWKS
SIASFFFIIGLIIGLFLVLRVGIHLCIKLKHTKKRQIYTDIEMNRLGKDIQHSGGRMSKGEELFTGVVPILVELDGDVNG
HKFSVSGEGEGDATYGKLTLKFICTTGKLPVPWPTLVTTLTYGVQCFSRYPDHMKQHDFFKSAMPEGYVQERTIFF
KDDGNYKTRAEVKFEGDTLVNRIELKGIDFKEDGNILGHKLEYNYNSHNVYIMADKQKNGIKVNFKIRHNIEDGSV
QLADHYQQNTPIGDGPVLLPDNHYLSTQSALSKDPNEKRDHMVLLEFVTAAGITHGMDELYK*

Annotation: CD47-Ecto; CD8 hinge; truncated VSVG (tVSVG); eGFP

Coding sequences of CD47-Ecto-tVSVG-GFP

ATGTGGCCCCTGGTGGCTGCCCTGCTGCTGGGAAGCGCCTGTTGCGGATCTGCTCAGCTGCTGTTCAATAAGA
CTAAGAGTGTGGAGTTTACATTCTGCAACGACACCGTGGTCATCCCATGTTTTGTGACAAACATGGAGGCCCA
GAATACCACAGAGGTGTACGTGAAGTGGAAGTTTAAGGGCAGGGACATCTATACCTTCGATGGCGCCCTGAA
CAAGAGCACAGTGCCCACCGACTTCAGCTCCGCCAAGATCGAGGTGTCTCAGCTGCTGAAGGGCGATGCCAG
CCTGAAGATGGACAAGTCTGATGCCGTGAGCCACACAGGCAATTACACCTGCGAGGTGACAGAGCTGACCAG
GGAGGGCGAGACAATCATCGAGCTGAAGTATCGCGTGGTGAGCTGGTTTTCCCCTAACGAGAATACCACAAC
CCCAGCACCCCGGCCCCCTACACCTGCACCAACCATCGCCTCCCAGCCACTGTCTCTGCGGCCTGAGGCCTGC
AGACCAGCAGCAGGAGGAGCAGTGCACACAAGAGGCCTGGACTTCGCCTGTGATGAGCACCCACACATCCA
GGACGCAGCCAGCCAGCTGCCTGACGATGAGTCCCTGTTCTTTGGCGATACCGGCCTGTCCAAGAATCCCATC
GAGCTGGTGGAGGGCTGGTTTTCTAGCTGGAAGTCCTCTATCGCCTCTTTCTTTTTCATCATCGGCCTGATCAT
CGGCCTGTTCCTGGTGCTGAGAGTGGGCATCCACCTGTGCATCAAGCTGAAGCATACTAAGAAGCGACAGAT
TTACACTGACATTGAGATGAACCGACTGGGAAAAGATATCCAGCACAGTGGCGGCCGCATGAGCAAGGGCG
AGGAGCTGTTCACCGGGGTGGTGCCCATCCTGGTCGAGCTGGACGGCGACGTAAACGGCCACAAGTTCAGC
GTGTCCGGCGAGGGCGAGGGCGATGCCACCTACGGCAAGCTGACCCTGAAGTTCATCTGCACCACCGGCAA
GCTGCCCGTGCCCTGGCCCACCCTCGTGACCACCCTGACCTACGGCGTGCAGTGCTTCAGCCGCTACCCCGAC
CACATGAAGCAGCACGACTTCTTCAAGTCCGCCATGCCCGAAGGCTACGTCCAGGAGCGCACCATCTTCTTCA
AGGACGACGGCAACTACAAGACCCGCGCCGAGGTGAAGTTCGAGGGCGACACCCTGGTGAACCGCATCGAG
CTGAAGGGCATCGACTTCAAGGAGGACGGCAACATCCTGGGGCACAAGCTGGAGTACAACTACAACAGCCA
CAACGTCTATATCATGGCCGACAAGCAGAAGAACGGCATCAAGGTGAACTTCAAGATCCGCCACAACATCGA
GGACGGCAGCGTGCAGCTCGCCGACCACTACCAGCAGAACACCCCCATCGGCGACGGCCCCGTGCTGCTGCC
CGACAACCACTACCTGAGCACCCAGTCCGCCCTGAGCAAAGACCCCAACGAGAAGCGCGATCACATGGTCCT
GCTGGAGTTCGTGACCGCCGCCGGGATCACTCACGGCATGGACGAGCTGTACAAGTAA

3. hTansferrin-tVSVG-cogGFP

Coded protein (1085 aa)

MRLAVGALLVCAVLGLCLAVPDKTVRWCAVSEHEATKCQSFRDHMKSVIPSDGPSVACVKKASYLDCIRAIAANE
ADAVTLDAGLVYDAYLAPNNLKPVVAEFYGSKEDPQTFYYAVAVVKKDSGFQMNQLRGKKSCHTGLGRSAGWNI
PIGLLYCDLPEPRKPLEKAVANFFSGSCAPCADGTDFPQLCQLCPGCGCSTLNQYFGYSGAFKCLKDGAGDVAFVK
HSTIFENLANKADRDQYELLCLDNTRKPVDEYKDCHLAQVPSHTVVARSMGGKEDLIWELLNQAQEHFGKDKSKE
FQLFSSPHGKDLLFKDSAHGFLKVPPRMDAKMYLGYEYVTAIRNLREGTCPEAPTDECKPVKWCALSHHERLKCDE
WSVNSVGKIECVSAETTEDCIAKIMNGEADAMSLDGGFVYIAGKCGLVPVLAENYNKSDNCEDTPEAGYFAVAVV
KKSASDLTWDNLKGKKSCHTAVGRTAGWNIPMGLLYNKINHCRFDEFFSEGCAPGSKKDSSLCKLCMGSGLNLCE
PNNKEGYYGYTGAFRCLVEKGDVAFVKHQTVPQNTGGKNPDPWAKNLNEKDYELLCLDGTRKPVEEYANCHLAR
APNHAVVTRKDKEACVHKILRQQQHLFGSNVTDCSGNFCLFRSETKDLLFRDDTVCLAKLHDRNTYEKYLGEEYVK
AVGNLRKCSTSSLLEACTFRRPTTTPAPRPPTPAPTIASQPLSLRPEACRPAAGGAVHTRGLDFACDEHPHIQDAAS
QLPDDESLFFGDTGLSKNPIELVEGWFSSWKSSIASFFFIIGLIIGLFLVLRVGIHLCIKLKHTKKRQIYTDIEMNRLGKM
ESDESGLPAMEIECRITGTLNGVEFELVGGGEGTPKQGRMTNKMKSTKGALTFSPYLLSHVMGYGFYHFGTYPSG
YENPFLHAINNGGYTNTRIEKYEDGGVLHVSFSYRYEAGRVIGDFKVVGTGFPEDSVIFTDKIIRSNATVEHLHPMG
DNVLVGSFARTFSLRDGGYYSFVVDSHMHFKSAIHPSILQNGGPMFAFRRVEELHSNTELGIVEYQHAFKTPIAFAR
SRAQSSNSAVDGTAGPGSTGSR*

Annotation: Human Transferrin; CD8 hinge; truncated VSVG (tVSVG); cogGFP

Coding sequences of hTransferrin-tVSVG-GFP

atgaggctcgccgtgggagccctgctggtctgcgccgtcctggggctgtgtctggctgtccctgataaaactgtgagatggtgtgcagtgtcgga
gcatgaggccactaagtgccagagtttccgcgaccatatgaaaagcgtcattccatccgatggtcccagtgttgcttgtgtgaagaaagcctcct
accttgattgcatcagggccattgcggcaaacgaagcggatgctgtgacactggatgcaggtttggtgtatgatgcttacttggctcccaataac
ctgaagcctgtggtggcagagttctatgggtcaaaagaggatccacagactttctattatgctgttgctgtggtgaagaaggatagtggcttcca
gatgaaccagcttcgaggcaagaagtcctgccacacgggtctaggcaggtccgctgggtggaacatccccataggcttactttactgtgacttac
ctgagccacgtaaacctcttgagaaagcagtggccaatttcttctcgggcagctgtgccccttgtgcggatgggacggacttcccccagctgtgtc
aactgtgtccagggtgtggctgctccacccttaaccaatacttcggctactcgggagccttcaagtgtctgaaggatggtgctggggatgtggcct
ttgtcaagcactcgactatatttgagaacttggcaaacaaggctgacagggaccagtatgagctgctttgcctagacaacacccggaagccggt
agatgaatacaaggactgccacttggcccaggtcccttctcataccgtcgtggcccgaagtatgggcggcaaggaggacttgatctgggagctt
ctcaaccaggcccaggaacattttggcaaagacaaatcaaaagaattccaactattcagctctcctcatgggaaggacctgctgtttaaggact
ctgcccacgggtttttaaaagtcccccaaggatggatgccaagatgtacctgggctatgagtatgtcactgccatccggaatctacgggaaggc
acatgcccagaagccccaacagatgaatgcaagcctgtgaagtggtgtgcgctgagccaccacgagaggctcaagtgtgatgagtggagtgtt
aacagtgtagggaaaatagagtgtgtatcagcagagaccaccgaagactgcatcgccaagatcatgaatggagaagctgatgccatgagctt
ggatggagggtttgtctacatagcgggcaagtgtggtctggtgcctgtcttggcagaaaactacaataagagcgataattgtgaggatacacca
gaggcagggtattttgctgtagcagtggtgaagaaatcagcttctgacctcacctgggacaatctgaaaggcaagaagtcctgccatacggcag
ttggcagaaccgctggctggaacatccccatgggcctgctctacaataagatcaaccactgcagatttgatgaatttttcagtgaaggttgtgccc
ctgggtctaagaaagactccagtctctgtaagctgtgtatgggctcaggcctaaacctgtgtgaacccaacaacaaagagggatactacggcta
cacaggcgctttcaggtgtctggttgagaagggagatgtggcctttgtgaaacaccagactgtcccacagaacactgggggaaaaaaccctgat
ccatgggctaagaatctgaatgaaaaagactatgagttgctgtgccttgatggtaccaggaaacctgtggaggagtatgcgaactgccacctgg
ccagagccccgaatcacgctgtggtcacacggaaagataaggaagcttgcgtccacaagatattacgtcaacagcagcacctatttggaagca
acgtaactgactgctcgggcaacttttgtttgttccggtcggaaaccaaggaccttctgttcagagatgacacagtatgtttggccaaacttcatg
acagaaacacatatgaaaaatacttaggagaagaatatgtcaaggctgttggtaacctgagaaaatgctccacctcatcactcctggaagcctg
cactttccgtagacctaccacgacgccagcgccgcgaccaccaacaccggcgcccaccatcgcgtcgcagcccctgtccctgcgcccagaggcg
tgccggccagcggcggggggcgcagtgcacacgagggggctggacttcgcctgtgatgaacatcctcacattcaagacgctgcttcgcaacttc

ctgatgatgagagtttatttttggtgatactgggctatccaaaaatccaatcgagcttgtagaaggttggttcagtagttggaaaagctctattgc
ctctttttctttatcatagggttaatcattggactattcttggttctccgagttggtatccatctttgcattaaattaaagcacaccaagaaaagaca
gatttatacagacatagagatgaaccgacttggaaagatggagagcgacgagagcggcctgcccgccatggagatcgagtgccgcatcaccg
gcaccctgaacggcgtggagttcgagctggtgggcggcggagagggcacccccaagcagggccgcatgaccaacaagatgaagagcaccaa
aggcgccctgaccttcagcccctacctgctgagccacgtgatgggctacggcttctaccacttcggcacctaccccagcggctacgagaacccct
tcctgcacgccatcaacaacggcggctacaccaacacccgcatcgagaagtacgaggacggcggcgtgctgcacgtgagcttcagctaccgct
acgaggccggccgcgtgatcggcgacttcaaggtggtgggcaccggcttccccgaggacagcgtgatcttcaccgacaagatcatccgcagca
acgccaccgtggagcacctgcaccccatgggcgataacgtgctggtgggcagcttcgcccgcaccttcagcctgcgcgacggcggctactacag
cttcgtggtggacagccacatgcacttcaagagcgccatccaccccagcatcctgcagaacgggggccccatgttcgccttccgccgcgtggag
gagctgcacagcaacaccgagctgggcatcgtggagtaccagcacgccttcaagacccccatcgccttcgccagatcccgcgctcagtcgtcca
attctgccgtggacggcaccgccggacccggctccaccggatctcgctaa

4. **mNCAM-tVSVG-GFP**

Coded protein (618 aa)

MLRTKDLIWTLFFLGTAVSLQVDIVPSQGEISVGESKFFLCQVAGDAKDKDISWFSPNGEKLSPNQQRISVVWNDD
DSSTLTIYNANIDDAGIYKCVVTAEDGTQSEATVNVKIFQKLMFKNAPTPQEFKEGEDAVIVCDVVSSLPPTIIWKHK
GRDVILKKDVRFIVLSNNYLQIRGIKKTDEGTYRCEGRILARGEINFKDIQVIVNVPPTVQARQSIVNATANLGQSVTL
VCDADGFPEPTMSWTKDGEPIENEEEDDEKHIFSDDSSELTIRNVDKNDEAEYVCIAENKAGILGDTGLSKNPIELV
EGWFSSWKSSIASFFFIIGLIIGLFLVLRVGIHLCIKLKHTKKRQIYTDIEMNRLGKESDESGLPAMEIECRITGTLNGVE
FELVGGGEGTPKQGRMTNKMKSTKGALTFSPYLLSHVMGYGFYHFGTYPSGYENPFLHAINNGGYTNTRIEKYED
GGVLHVSFSYRYEAGRVIGDFKVVGTGFPEDSVIFTDKIIRSNATVEHLHPMGDNVLVGSFARTFSLRDGGYYSFVV
DSHMHFKSAIHPSILQNGGPMFAFRRVEELHSNTELGIVEYQHAFKTPIAFARSRAQSSNSAVDGTAGPGSTGSR*

Annotation: Mouse NCAM; truncated VSVG (tVSVG); cogGFP

Coding sequences of mNCAM-tVSVG-GFP

ATGCTGCGAACTAAGGATCTCATCTGGACTTTGTTTTTCCTGGGAACTGCAGTTTCCCTGCAGGTAGATATTGT
TCCCAGCCAAGGAGAAATCAGCGTTGGAGAGTCCAAATTCTTCCTGTGTCAAGTGGCAGGAGATGCCAAAGA
TAAGGACATCTCCTGGTTCTCCCCCAATGGGGAGAAGCTGAGCCCAAACCAGCAGCGGATCTCAGTGGTGTG
GAATGATGACGACTCCTCTACCCTCACCATCTACAACGCCAACATCGACGATGCCGGCATATACAAGTGTGTG
GTCACGGCTGAGGACGGCACCCAGTCTGAGGCCACTGTCAACGTGAAGATCTTCCAGAAGCTCATGTTCAAG
AATGCACCAACCCCACAGGAGTTTAAGGAAGGGGAGGATGCTGTGATTGTCTGTGATGTGGTCAGCTCCCTG
CCTCCAACCATCATCTGGAAACACAAAGGCCGAGATGTCATTCTGAAAAAAGACGTCCGGTTCATAGTCCTGT
CCAACAACTACCTGCAGATCAGGGGCATCAAGAAAACAGATGAGGGTACTTACCGCTGTGAGGGCAGGATCC
TGGCCCGCGGGGAAATCAACTTCAAGGACATTCAGGTCATTGTGAATGTACCACCCACTGTCCAGGCCAGAC
AGAGCATCGTGAATGCCACTGCCAACCTGGGCCAGTCTGTCACCCTGGTGTGTGATGCCGATGGCTTCCCAGA
GCCCACCATGAGCTGGACAAAGGATGGGGAACCCATAGAGAACGAGGAGGAAGATGACGAGAAGCACATC
TTCAGTGATGACAGCTCCGAGCTGACCATCAGGAATGTGGATAAAAACGACGAGGCCGAATACGTCTGCATC
GCAGAGAACAAGGCTGgaattcttggtgatactgggctatccaaaaatccaatcgagcttgtagaaggttggttcagtagttggaaaag
ctctattgcctctttttctttatcatagggttaatcattggactattcttggttctccgagttggtatccatctttgcattaaattaaagcacaccaag
aaaagacagatttatacagacatagagatgaaccgacttggaaaggagagcgacgagagcggcctgcccgccatggagatcgagtgccgcat
caccggcaccctgaacggcgtggagttcgagctggtgggcggcggagagggcacccccaagcagggccgcatgaccaacaagatgaagagc

accaaaggcgccctgaccttcagcccctacctgctgagccacgtgatgggctacggcttctaccacttcggcacctaccccagcggctacgaga
acccccttcctgcacgccatcaacaacggcggctacaccaacacccgcatcgagaagtacgaggacggcggcgtgctgcacgtgagcttcagct
accgctacgaggccggccgcgtgatcggcgacttcaaggtggtgggcaccggcttccccgaggacagcgtgatcttcaccgacaagatcatccg
cagcaacgccaccgtggagcacctgcaccccatgggcgataacgtgctggtgggcagcttcgcccgcaccttcagcctgcgcgacggcggctac
tacagcttcgtggtggacagccacatgcacttcaagagcgccatccaccccagcatcctgcagaacgggggccccatgttcgccttccgccgcgt
ggaggagctgcacagcaacaccgagctgggcatcgtggagtaccagcacgccttcaagacccccatcgccttcgccagatcccgcgctcagtc
gtccaattctgccgtggacggcaccgccggacccggctccaccggatctcgctaa
