# Supplementary material for: Dual-Omics Approach Unveils Novel Perspective on the Quality Control of Genetically Engineered Exosomes
Source: Pharmaceutics. 2024 Jun 18;16(6):824. doi: 10.3390/pharmaceutics16060824 (PMC11207238; doi:10.3390/pharmaceutics16060824)
Supplement: Supplementary file 1 [file pharmaceutics-16-00824-s001.zip › Table S1.pdf]

Table S1: 131 Cellular Pathways Regulated by miRNAs

Number	Name of Pathways
1	Signal transduction
2	Cell communication
3	Regulation of nucleobase metabolism
4	Metabolism
5	Transport
6	Energy pathways
7	Protein metabolism
8	Cell growth and/or maintenance
9	Immune response
10	Apoptosis
11	Regulation of gene expression
12	Cell adhesion
13	Regulation of cell cycle
14	Transcription
15	Ion transport
16	Cell proliferation
17	Lipid metabolism
18	DNA repair
19	Anti-apoptosis
20	Regulation of cell growth
21	Cell differentiation
22	regulation of signal transduction

23	Cell cycle
24	Regulation of cell proliferation
25	Cell motility
26	Protein modification
27	Vesicle mediated transport
28	Cytoskeleton organization
29	Regulation of translation
30	Cell-Cell signaling
31	Cell migration
32	Cell-Cell adhesion
33	RNA metabolism
34	Fatty acid metabolism
35	protein transport
36	protein folding
37	cell organization and biogenesis
38	carbohydrate metabolism
39	regulation of immune response
40	cell surface receptor linked signal transduction
41	embryonic development
42	chromosome segregation
43	cell development
44	organogenesis
45	neurogenesis
46	DNA replication

47	regulation of exocytosis
48	Regulation of metabolism
49	Proteolysis and peptidolysis
50	Peptide metabolism
51	Cell death
52	Hemopoiesis
53	Nucleobase, nucleoside, nucleotide, and nucleic acid transport
54	G-protein coupled receptor protein signaling pathway
55	Morphogenesis
56	Regulation of cellular process
57	Development
58	Electron transport
59	Endosome transport
60	Protein targeting
61	Regulation of development
62	Mitosis
63	Microtubule-based process
64	Lipid transport
65	Neurotransmitter transport
66	Regulation of enzyme activity
67	Cell fate commitment
68	Vesicle docking
69	Cytokine and chemokine mediated signaling pathway
70	Synaptic transmission

71	Cell recognition
72	Skeletal development
73	Muscle development
74	Amino acid and derivative metabolism
75	Chromosome organization and biogenesis
76	Wound healing
77	Signal complex formation
78	Steroid hormone receptor signaling pathway
79	Steroid metabolism
80	Amino acid transport
81	Cellular morphogenesis during differentiation
82	Gene silencing
83	Regulation of transport
84	Intracellular signaling cascade
85	Mitochondrion organization and biogenesis
86	CAMP-mediated signaling
87	Cellular morphogenesis during differentiation
88	Vasodilation
89	Inflammatory response
90	Bone remodeling
91	Negative regulation of enzyme activity
92	Regulation of endocytosis
93	Drug metabolism
94	Innate immune response

95	Synaptic vesicle transport
96	Cell-matrix adhesion
97	Cytoskeletal anchoring
98	Reproduction
99	Regulation of physiological processes
100	Synapse organization and biogenesis
101	Immune cell migration
102	Muscle contraction
103	Calcium-mediated signaling
104	Ribosome biogenesis and assembly
105	Cellular defense response
106	Protein localization
107	Glycoprotein metabolism
108	Vesicle organization and biogenesis
109	Regulation of hormone secretion
110	Learning and/or memory
111	Phosphoinositide-mediated signaling
112	Regulation of circadian rhythm
113	G-protein signaling, coupled to cyclic nucleotide second messenger
114	Antigen receptor-mediated signaling pathway
115	Peroxisome organization and Biogenesis
116	Cell homeostasis
117	Antigen presentation
118	Lymphocyte activation

119	Enzyme linked receptor protein signaling pathway
120	Neurotransmitter metabolism
121	DNA metabolism
122	RNA localization
123	Hormone metabolism
124	Vitamin metabolism
125	Transmembrane receptor protein tyrosine kinase signaling pathway
126	Extracellular structure organization and biogenesis
127	Lymphocyte proliferation
128	Plasma membrane organization and biogenesis
129	Vitamin/cofactor transport
130	Carbohydrate mediated signaling
131	Regulation of viral life cycle
