# Supplementary material for: Dual-Omics Approach Unveils Novel Perspective on the Quality Control of Genetically Engineered Exosomes
Source: Pharmaceutics. 2024 Jun 18;16(6):824. doi: 10.3390/pharmaceutics16060824 (PMC11207238; doi:10.3390/pharmaceutics16060824)
Supplement: Supplementary file 1 [file pharmaceutics-16-00824-s001.zip › Table S2.pdf]

Table S2: 52 Cellular Pathways Regulated by Proteins

Number	Name of Pathways
1	Signal Transduction
2	Protein Metabolism
3	Cell Communication
4	Regulation of Nucleobase Metabolism
5	Metabolism
6	Energy Pathways
7	Cell growth and/or Maintenance
8	Transport
9	Immune response
10	Apoptosis
11	Regulation of cell cycle
12	Protein folding
13	Cell cycle
14	RNA metabolism
15	Cell proliferation
16	DNA repair
17	Regulation of gene expression
18	Carbohydrate metabolism
19	Cell motility
20	Cytoskeletal anchoring
21	Cytoskeletal organization
22	Regulation of cell proliferation

23	Cell adhesion
24	Transcription
25	Anti-apoptosis
26	Cell migration
27	Chromosome organization
28	Protein targeting
29	Immune cell migration
30	RNA localization
31	Vesicle-mediated transport
32	Mitochondrion organization
33	Regulation of cell growth
34	Regulation of signal transduction
35	TRNA aminoacylation
36	Cell surface receptor linked signal transduction
37	Glycosaminoglycan metabolism
38	Purine salvage
39	Lipid metabolism
40	Regulation of immune response
41	Peptide metabolism
42	Morphogenesis
43	Mitosis
44	Cell maturation
45	Neurogenesis

46	Lipoprotein metabolism
47	Carbohydrate mediated signaling
48	Cell-Cell Signalling
49	Proteolysis and peptidolysis
50	Organogenesis
51	Regulation of translation
52	DNA replication
