# Supplementary material for: Dual-Omics Approach Unveils Novel Perspective on the Quality Control of Genetically Engineered Exosomes
Source: Pharmaceutics. 2024 Jun 18;16(6):824. doi: 10.3390/pharmaceutics16060824 (PMC11207238; doi:10.3390/pharmaceutics16060824)
Supplement: Supplementary file 1 [file pharmaceutics-16-00824-s001.zip › Table S3.pdf]

Table S3. Summary of 99 Protein Contaminants and Their Relative Abundance in Control and Engineered Exosomes

Number	Accession	Species	Protein Name	Description	Control Abundance	CD47 Abundance	CD47tVSVG Abundance	RVG-TN Abundance	RVG-NCAM Abundance
1	P02769	Bos taurus	Albumin	SWISS-PROT:P02769 (Bos taurus) Bovine serum albumin precursor	29162416226	26208720650	24932069778	22018097356	20877964271
2	ENSEMBL:ENSBTAP00000024146	Bos taurus	Alpha-2-macroglobulin	(Bos taurus) similar to alpha-2-macroglobulin isoform 1	13190873935	11214638431	11902375520	8593073311	9856293021
3	P15497	Bos taurus	Apolipoprotein A-I	SWISS-PROT:P15497 (Bos taurus) Apolipoprotein A-I precursor	9093516982	9794427645	7543557989	9761704066	7794380539
4	Q3KUS7	Bos taurus	C3/C5 covertase	TREMBL:Q3KUS7 (Bos taurus) Complement factor B	5568670030	4926331207	3448389430	4850782388	3648781631
5	Q2UVX4	Bos taurus	Complement C3	SWISS-PROT:Q2UVX4 (Bos taurus) Complement C3 precursor	5546194605	4660267109	4481488676	3521915263	4195946844
6	Q0IIK2	Bos taurus	Serotransferrin	TREMBL:Q0IIK2 (Bos taurus) Transferrin	4650675262	4759933975	3072477172	5627152526	3100399543
7	ENSEMBL:ENSBTAP00000024462	Bos taurus	Protein retired from ENSEMBL database (no info found)	(Bos taurus) 47 kDa protein	2759026544	2601105774	2642219905	2445853901	2452807066
8	Q3SX09	Bos taurus	Hemoglobin fetal subunit beta	TREMBL:Q3SX09 (Bos taurus) similar to HBG protein	2169782353	1815580723	2065417363	1672321373	1829579269

9	P34955	Bos taurus	Alpha-1-antiproteinase	SWISS-PROT:P34955 (Bos taurus) Alpha-1-antiproteinase precursor	2058696282	1904586987	2054990136	1638448193	1499884998
10	Q1RMN8	Bos taurus	Immunoglobulin light chain	TREMBL:Q1RMN8 (Bos taurus) Similar to Immunoglobulin lambda-like polypeptide 1	1957440643	1837567472	1283469177	1660096517	1358123778
11	Q3SZ57	Bos taurus	Alpha-fetoprotein	SWISS-PROT:Q3SZ57 (Bos taurus) Alpha-fetoprotein precursor	1154602290	960079916.8	925988097.2	752030539.8	838590576.6
12	P01966	Bos taurus	Hemoglobin subunit alpha	SWISS-PROT:P01966 (Bos taurus) Hemoglobin subunit alpha	1044139767	871489947.8	890625895.5	847837098.4	799353119.8
13	Q1RMK2	Bos taurus	Deleted	TREMBL:Q1RMK2 (Bos taurus) IGHM protein	967628396.3	849777270.4	802979723.7	717287995.3	838906239.8
14	P41361	Bos taurus	Antithrombin-III	SWISS-PROT:P41361 (Bos taurus) Antithrombin-III precursor	761335033.1	677325472	529118103.5	620370212.8	498272636.8
15	P12763	Bos taurus	Alpha-2-HS-glycoprotein	SWISS-PROT:P12763 (Bos taurus) Alpha-2-HS-glycoprotein precursor	657551912.9	551739890.6	617665263.8	394699359.2	435368458.7
16	Q3SZV7	Bos taurus	Hemopexin	TREMBL:Q3SZV7 (Bos taurus) Similar to hemopexin	648354038.3	787307622.6	407233635.7	813875204.5	499794787.3
17	Q9TRI1	Bos taurus	Inter alpha trypsin inhibitor HC2 component homolog	TREMBL:Q9TRI1 (Bos taurus) similar to inter-alpha-trypsin inhibitor heavy chain2	624699421.2	533972706.2	500662548.9	479290860.7	407172963.2

18	ENSEMBL:ENSBTAP00000007350	Bos taurus	Complement Component 4A	(Bos taurus) similar to Complement C4-A precursor	521023879.6	514161825.5	551822502.8	407782205.8	505599912.5
19	Q05B55	Bos taurus	Ig-like domain-containing protein (from Interpro database)	TREMBL:Q05B55 (Bos taurus) Similar to Ig kappa chain C region	403575367.3	302630214.1	183952080.9	236726117.9	253415983.6
20	P02070	Bos taurus	Hemoglobin subunit beta	SWISS-PROT:P02070 (Bos taurus) Hemoglobin subunit beta	394731719.5	500194677.4	431528962.7	468196526.2	435674766.3
21	ENSEMBL:ENSBTAP00000037665	Bos taurus	Protein retired from ENSEMBL database (no info found)	(Bos taurus) similar to Pregnancy zone protein, partial	387356964.4	330498698.9	322908209.6	239102208.7	278041568.9
22	Q3T052	Bos taurus	Inter-alpha-trypsin inhibitor heavy chain H4	TREMBL:Q3T052;Q5EA67 (Bos taurus) Inter-alpha (Globulin) inhibitor H4	345409681.8	314559056.1	314515535.5	284632796.6	247430827.2
23	P06868	Bos taurus	Plasminogen	SWISS-PROT:P06868 (Bos taurus) Plasminogen precursor	300868928.6	193683098.9	208363486.2	137469265.5	160554205.3
24	Q28085	Bos taurus	Complement factor H	SWISS-PROT:Q28085 (Bos taurus) Complement factor H precursor	297952674.5	228795680.7	232411220.6	196914699.9	207528949.8
25	ENSEMBL:ENSBTAP00000014147	Bos taurus	Protein retired from ENSEMBL database (no info found)	(Bos taurus) 12 kDa protein	281668688.8	233119683.1	217491590.1	189389747.7	195657428.1
26	A2I7N0	Bos taurus	Serpin A3-4	TREMBL:A2I7N0;Q28922;Q3ZEJ6 (Bos taurus) SERPINA3-4	273359245.1	268010124.7	229862471.6	226241256.2	197972958.8

27	Q3Y5Z3	Bos taurus	Adiponectin	SWISS-PROT:Q3Y5Z3 (Bos taurus) Adiponectin precursor	256812593.1	232205181.8	251627329.5	187424703.7	210499157.3
28	Q1A7A4	Bos taurus	Complement component C5a	TREMBL:Q1A7A4 (Bos taurus) similar to complement component C5	226255076.2	190406375.4	159355782	136550613.3	137941337.1
29	REFSEQ:XP_001252647	Bos taurus	(from raw description): similar to endopin2B	(Bos taurus) similar to endopin 2B	218417764.2	193144289.4	157465664.3	168628131.7	130151629.7
30	P00735	Bos taurus	Prothrombin	SWISS-PROT:P00735 (Bos taurus) Prothrombin precursor (Fragment)	208293844.8	208085380	182735654.2	182575163.9	184406805.1
31	Q32PJ2	Bos taurus	Apolipoprotein A-IV	SWISS-PROT:Q32PJ2 (Bos taurus) Apolipoprotein A-IV precursor	191169281.8	203649104.2	165283520.5	196315423.8	173217990.6
32	P17690	Bos taurus	Beta-2-glycoprotein 1	SWISS-PROT:P17690 (Bos taurus) Beta-2-glycoprotein 1 precursor	182794593.3	172243362.3	133984280.8	145663040.2	117969211.9
33	Q0VCM5	Bos taurus	Inter-alpha-trypsin inhibitor heavy chain H1	TREMBL:Q0VCM5 (Bos taurus) Similar to Inter-alpha-trypsin inhibitor heavy chain H1	164713963.1	152540833.3	142574525.2	151151000.4	150816577.7
34	Q3SZH5	Bos taurus	Angiotensinogen	TREMBL:Q3SZH5 (Bos taurus) Similar to Angiotensinogen	152732900.9	130166240.5	153146155.8	127295737.9	119615048.1
35	Q3MHN2	Bos taurus	Complement component C9	SWISS-PROT:Q3MHN2 (Bos taurus) Complement	149973850.2	152022685.8	110451885.7	130516951.6	109709878.4

				component C9 precursor					
36	ENSEMBL:ENSBTAP00000032840	Bos taurus	Similar to alipoprotein B (from description)	(Bos taurus) similar to apolipopr40otein B, partial	147818456.5	113729911.3	133112591.4	87657862.13	103621455.3
37	Q0V8M9	Bos taurus	Inter-alpha-trypsin inhibitor heavy chain H3	TREMBL:Q0V8M9;Q9TRI0 (Bos taurus) similar to inter-alpha (globulin) inhibitor H3 isoform 2	143775183.7	126088712.4	125137582.4	106637638.5	109201880.5
38	A2I7N1	Bos taurus	Serpin A3-5	TREMBL:A2I7N1 (Bos taurus) SERPINA3-5	140178426.4	139583962.5	111074968.3	111821522.7	110990255.1
39	Q95121	Bos taurus	Pigment epithelium-derived factor	SWISS-PROT:Q95121 (Bos taurus) Pigment epithelium-derived factor precursor	132719621.6	102226132.5	122981619.2	91968005.87	73853326.51
40	Q3SX14	Bos taurus	Gelsolin	TREMBL:Q3SX14 (Bos taurus) Similar to Gelsolin	132090912.3	115588727	118282365.1	100802495.8	115756944.9
41	Q28194	Bos taurus	Thrombospondin-1	TREMBL:Q28194 (Bos taurus) Thrombospondin-1	118083150.3	82458492.72	70243236.5	63029647.05	55157932.42
42	ENSEMBL:ENSBTAP00000024466	Bos taurus	Protein retired from ENSEMBL database (no info found)	(Bos taurus) kDa protein	118078537.8	175426017	130962661	164275032.4	177612225.4
43	P81644	Bos taurus	Apolipoprotein A-II	SWISS-PROT:P81644 (Bos taurus) Apolipoprotein A-II precursor	112169169	86246885.35	98569640.78	83712317.51	82805573.32
44	ENSEMBL:ENSBTAP00000018574	Bos taurus	Serpin Family D member	(Bos taurus) 55 kDa protein	97007006.92	98212224.92	100511002	81571818.65	82574071.04
45	Q9N2I2	Bos taurus	Plasma serine protease inhibitor	SWISS-PROT:Q9N2I2 (Bos taurus)	95711270.01	89729083.21	43358018.06	71244795.02	42475184.87

				Plasma serine pr47otease inhib48itor precurs49or					
46	Q2KIH2	Bos taurus	Alipoprotein N	TREMBL:Q2KIH2;Q68RU0 (Bos taurus) Ovarian and testicular apolipoprotein N	82314606.22	75742909.48	66842649.63	74159221.25	61291550.61
47	Q9TTE1	Bos taurus	Serpin A3-1	SWISS-PROT:Q9TTE1 (Bos taurus) Endopin-1 precursor	79212100.23	86200797.04	42308300.79	52359921.17	41371171.43
48	ENSEMBL:ENSBTAP00000031900	Bos taurus	ceruloplasmin	(Bos taurus) 121 kDa protein	77831973.26	83440803.94	64316418.66	83899306.88	62241198.4
49	Q58D62	Bos taurus	Fetuin-B	SWISS-PROT:Q58D62 (Bos taurus) Fetuin-B precursor	77006613.59	71881418.05	81866727.92	66781274.5	62819366.58
50	Q32PI4	Bos taurus	Complement factor I	TREMBL:Q32PI4 (Bos taurus) Similar to complement factor I	75499733.03	68447308.78	49519010.17	64944986.07	45400715.27
51	Q29RQ1	Bos taurus	Complement component C7	SWISS-PROT:Q29RQ1 (Bos taurus) Complement component C7 precursor	71723300.5	49687904.2	62934602.29	37012719.86	47277197.76
52	Q2KIS7	Bos taurus	Tetranectin	SWISS-PROT:Q2KIS7 (Bos taurus) Tetranectin precursor	54839147.13	50815087.69	18501466.13	34069979.61	31269787.16
53	Q3ZBS7	Bos taurus	Vitronectin	TREMBL:Q3ZBS7 (Bos taurus) Vitronectin	53800535.7	59531442.28	62826760.88	45473516.32	52876546.99
54	ENSEMBL:ENSBTAP00000018229	Bos taurus	GC vitamin D binding protein	(Bos taurus) 54 kDa protein	51555898.37	91217445.84	103779564.4	185614613.6	165307452.7

55	ENSEMBL:ENSBTAP00000016046	Bos taurus	Fibulin-1 C (from descr)	(Bos taurus) similar to fibulin-1 C isoform 1	50246148.04	45737548.38	42482194.88	27184943.83	49742431.35
56	Q28065	Bos taurus	C4b-binding protein alpha chain	SWISS-PROT:Q28065 (Bos taurus) C4b-binding protein alpha chain precursor	45626185.19	35761851.48	33985315.31	29011399.64	31622024.21
57	P50448	Bos taurus	Factor XIIa inhibitor	SWISS-PROT:P50448 (Bos taurus) Factor XIIa inhibitor precursor	43666442.22	36027036.45	24313407.78	39027475.78	27600472.05
58	ENSEMBL:ENSBTAP00000031360	Bos taurus	Protein retired from ENSEMBL database (no info found)	(Bos taurus) 55 kDa protein	39766258.21	37874612.75	30721438.19	29412204.71	29917485.95
59	Q2KIF2	Bos taurus	Leucine-rich alpha-2-glycoprotein 1	TREMBL:Q2KIF2 (Bos taurus) Similar to leucine-rich alpha-2-glycoprotein 1	39420683.33	43304007.87	41951192.5	34703111.31	34530382.54
60	P28800	Bos taurus	Alpha-2-antiplasmin	SWISS-PROT:P28800 (Bos taurus) Alpha-2-antiplasmin precursor	37671130.81	42305109.42	49620982.83	42955038.08	40027073.5
61	P00978	Bos taurus	Protein AMBP	SWISS-PROT:P00978 (Bos taurus) AMBP protein precursor	35346799.38	44379464.73	34324234.5	38439768.8	33820625.16
62	ENSEMBL:ENSBTAP00000034412	Bos taurus	similar to C4b-binding protein alpha chain (from descr)	(Bos taurus) similar to C4b-binding protein alpha chain	34192057.16	29308479.53	25981833.23	20126119.25	24869518.18
63	P07224	Bos taurus	Vitamin K-dependent protein S	SWISS-PROT:P07224 (Bos taurus) Vitamin K-	29867247.7	28942735.86	30769915.78	24110751.95	23126139.77

				dependent protein S prec66ursor					
64	Q9TT36	Bos taurus	Thyroxine-binding globulin	SWISS-PROT:Q9TT36 (Bos taurus) Thyroxine-binding globulin precursor	28416256.43	29539152.78	30820124.56	22551342.86	28681790.73
65	Q3SZR3	Bos taurus	Alpha-1-acid glycoprotein	SWISS-PROT:Q3SZR3 (Bos taurus) Alpha-1-acid glycoprotein precursor	26203230.39	18382261.28	27910273.78	14579729.86	18733226.41
66	P02676	Bos taurus	Fibrinogen beta chain	SWISS-PROT:P02676 (Bos taurus) similar to Fibrinogen beta chain precursor	26053494.11	20471553.46	23963095.06	11098940.03	13869693.3
67	A2I7N3	Bos taurus	Serpin A3-7	TREMBL:A2I7N3;Q27984 (Bos taurus) SERPINA3-7	24485049.52	25741118.61	22179257.75	20101258.11	22202054.32
68	Q2KJC7	Bos taurus	Periostin	TREMBL:Q2KJC7;Q8HZM3 (Bos taurus) Periostin, osteoblast specific factor	24076622.1	26717835.57	22747902.19	19362658.12	20666215.99
69	P17697	Bos taurus	Clusterin	SWISS-PROT:P17697 (Bos taurus) Clusterin precursor	22309719.81	22094253.32	22575476.63	14122118.07	17772343.27
70	Q28107	Bos taurus	Coagulation factor V	SWISS-PROT:Q28107 (Bos taurus) Coagulation factor V precursor	21975972.2	15207840.58	18945882.76	13715398.14	15516635.98
71	ENSEMBL:ENSBTAP00000023402	Bos taurus	Serpin family A member	(Bos taurus) 46 kDa protein	21466906.74	23356078.54	26382223.59	22636524.56	23044651.36

72	ENSEMBL:ENSBTAP00000033053	Bos taurus	Error	(Bos taurus) 15 kDa protein	20456142.19	10704449.88	22346875.63	14613230.39	10574964.79
73	Q03247	Bos taurus	Apolipoprotein E	SWISS-PROT:Q03247 (Bos taurus) Apolipoprotein E precursor	19062043.09	17550729.1	18905981.09	11675177.94	11507637.23
74	Q05443	Bos taurus	Lumican	SWISS-PROT:Q05443 (Bos taurus) Lumican precursor	18164623.42	14579546.01	17555714.61	12812152.4	13189823.85
75	Q2KIG3	Bos taurus	Carboxypeptidase B2	TREMBL:Q2KIG3 (Bos taurus) Similar to carboxypeptidase B2	17260500.26	10187034.41	12442050.53	11493246.27	11290368.73
76	Q2KJ62	Bos taurus	Kininogen-1	TREMBL:Q2KJ62 (Bos taurus) KNG protein	17017667.89	18166610.57	15932357.31	14469312.14	12363061.23
77	P01030	Bos taurus	Complement C4	SWISS-PROT:P01030 (Bos taurus) similar to Complement C4-A precursor	16836996.77	18767609.59	20965022.46	18838903.61	23570874.55
78	Q2KJF1	Bos taurus	Alpha-1B-glycoprotein	TREMBL:Q2KJF1 (Bos taurus) Alpha-1-B glycoprotein	15590160.84	12313154.76	13341407.91	8281876.276	8515505.057
79	Q3MHH8	Bos taurus	alpha-amylase	TREMBL:Q3MHH8 (Bos taurus) Amylase, alpha 2B; pancreatic	14194296.16	13379592.84	13011474.69	8833527.922	8580073.483
80	ENSEMBL:ENSBTAP00000006074	Bos taurus	Cartilage Oligomeric matrix protein	(Bos taurus) 81 kDa protein	11775567.65	8317144.484	9383807.758	8732991.769	6016531.07
81	P02672	Bos taurus	Fibrinogen alpha chain	SWISS-PROT:P02672 (Bos taurus) Fibrinogen alpha chain precursor	11423445.89	8621316.513	7456891.875	5198806.009	7817487.479
82	Q2HJF0	Bos taurus	Serotransferrin-like	TREMBL:Q2HJF0 (Bos taurus)	11173101.89	9952154.682	6752029.656	11310436.35	3020917.812

				Similar to Serotransferrin					
83	Q3MHN5	Bos taurus	Vitamin D-binding protein	SWISS-PROT:Q3MHN5 (Bos taurus) Vitamin D-binding protein precursor	10054342.32	10697608.84	17552157.59	30099146.66	22047930.89
84	ENSEMBL:ENSBTAP00000038329	Bos taurus	Serpin family	(Bos taurus) 9 kDa protein	9731236.934	7247447.264	4819999.5	4081362.611	4966723.27
85	Q95M17	Bos taurus	Acidic mammalian chitinase	SWISS-PROT:Q95M17 (Bos taurus) Acidic mammalian chitinase precursor	9412364.911	9370800.961	9518155.813	8351198.777	7741266.5
86	P02777	Bos taurus	Platelet factor 4	SWISS-PROT:P02777 (Bos taurus) similar to Platelet factor 4	5691584.664	424150.3986	432213.7188	965678.6609	Not Found
87	Q2TBQ1	Bos taurus	Coagulation factor XIII B chain	TREMBL:Q2TBQ1 (Bos taurus) Coagulation factor XIII, B polypeptide	5215081.698	5300575.135	3614039.703	3371119.46	3103946.505
88	ENSEMBL:ENSBTAP00000013050	Bos taurus	Serpin family A member	(Bos taurus) hypothetical protein	3951911.23	3321063.762	4725843.531	3194303.618	4195944.35
89	ENSEMBL:ENSBTAP00000011227	Bos taurus	Protein retired from ENSEMBL database (no info found)	(Bos taurus) 15 kDa protein	3517183.241	3576233.841	4703463.688	4170655.894	2555176.946
90	Q2KJ83	Bos taurus	Carboxypeptidase N catalytic chain	TREMBL:Q2KJ83 (Bos taurus) Similar to Carboxypeptidase N catalytic chain	3346816.862	2260985.638	2938035.75	2373454.896	2097277.365
91	Q2KIT0	Bos taurus	Protein HP-20 homolog	TREMBL:Q2KIT0 (Bos taurus) Similar to collagen, type X, alpha 1	3219005.619	4187001.858	5896364.961	4455388.675	4128394.722

92	P02584	Bos taurus	Profilin-1	SWISS-PROT:P02584 (Bos taurus) Profilin-1	2221066.112	1220611.214	1403265.375	1178697.13	490034.8151
93	REFSEQ:XP_585019	Bos taurus	Similar to Afamin (from descr)	(Bos taurus) similar to afamin	1804600.682	1679558.318	401233.6563	290152.7081	2490168.106
94	P01045-1	Bos taurus	Isoform HMW of Kininogen-2	SWISS-PROT:P01045-1 (Bos taurus) Isoform HMW of Kininogen-2 precursor	682875.3297	604152.2904	587876.125	759272.2872	484176.5745
95	Q6T181	Bos taurus	Sex hormone-binding globulin	TREMBL:Q6T181;Q6T182 (Bos taurus) similar to sex hormone-binding globulin	550817.6025	445139.436	817764.375	290013.5961	871416.7016
96	ENSEMBL:ENSBTAP00000023055	Bos taurus	HGF Activator	(Bos taurus) 68 kDa protein	490231.864	630063.2407	697896.4063	1207101.732	301806.9044
97	Q3ZBD7	Bos taurus	Glucose-6-phosphate isomerase	SWISS-PROT:Q3ZBD7 (Bos taurus) Glucose-6-phosphate isomerase	332545.0747	812952.4997	582410.875	1415444.567	984727.7412
98	ENSEMBL:ENSBTAP00000025008	Bos taurus	Tubulin beta 1 class VI	(Bos taurus) hypothetical protein	256510.3024	Not Found	Not Found	Not Found	319664.5191
99	Q2KIU3	Bos taurus	Protein HP-25 homolog 2	TREMBL:Q2KIU3 (Bos taurus) Similar to C1q and tumor necrosis factor related protein 5	Not Found	Not Found	Found,Unquantified	Not Found	Not Found
