# Supplementary material for: Dual-Omics Approach Unveils Novel Perspective on the Quality Control of Genetically Engineered Exosomes
Source: Pharmaceutics. 2024 Jun 18;16(6):824. doi: 10.3390/pharmaceutics16060824 (PMC11207238; doi:10.3390/pharmaceutics16060824)
Supplement: Supplementary file 1 [file pharmaceutics-16-00824-s001.zip › Figures-S1-S3.pptx]

## Slide 1
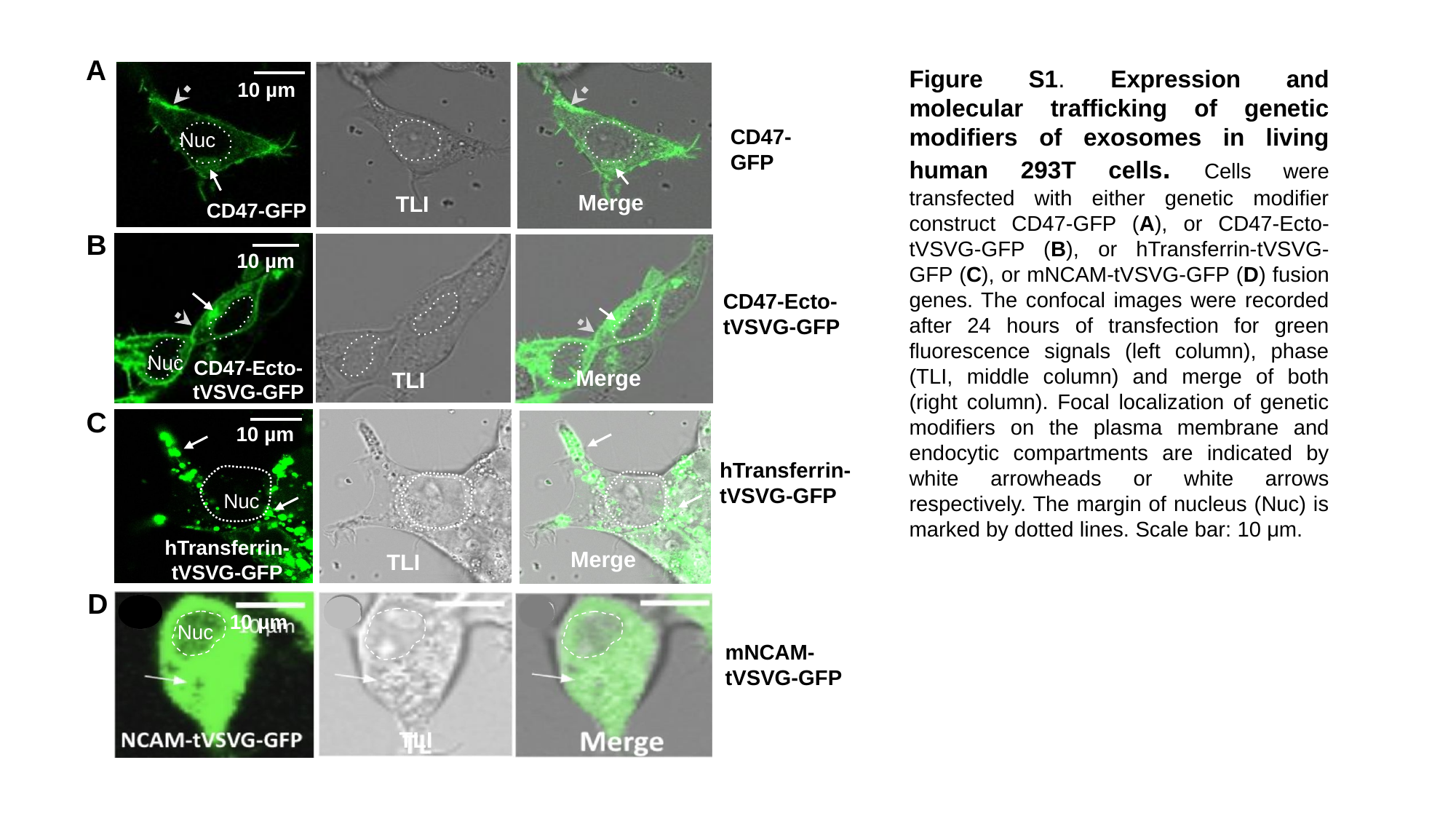

A
10 µm
CD47Ecto-tVSVG-eGFP TLI Overlay
Merge
TLI
CD47-GFP
10 µm
CD47-Ecto-tVSVG-GFP
Merge
TLI
10 µm
hTransferrin-tVSVG-GFP
Merge
TLI
10 µm
TLI
Nuc
CD47-GFP
B
CD47-Ecto-tVSVG-GFP
Nuc
C
hTransferrin-tVSVG-GFP
Nuc
D
Nuc
mNCAM-tVSVG-GFP
Figure S1. Expression and molecular trafficking of genetic modifiers of exosomes in living human 293T cells. Cells were transfected with either genetic modifier construct CD47-GFP (A), or CD47-Ecto-tVSVG-GFP (B), or hTransferrin-tVSVG-GFP (C), or mNCAM-tVSVG-GFP (D) fusion genes. The confocal images were recorded after 24 hours of transfection for green fluorescence signals (left column), phase (TLI, middle column) and merge of both (right column). Focal localization of genetic modifiers on the plasma membrane and endocytic compartments are indicated by white arrowheads or white arrows respectively. The margin of nucleus (Nuc) is marked by dotted lines. Scale bar: 10 μm.

## Slide 2
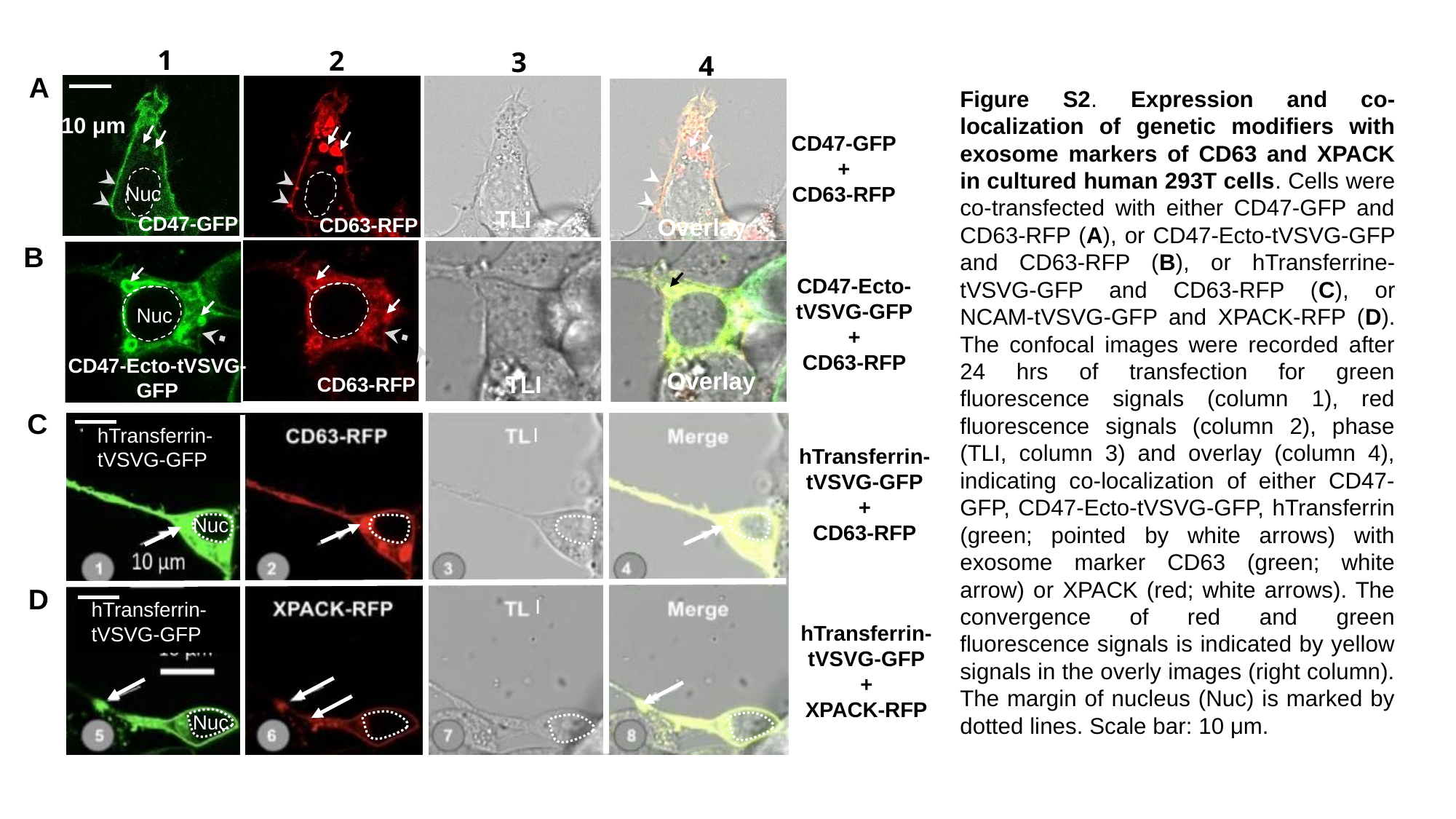

1
2
3
4
A
10 μm
Nuc
TLI
CD47-GFP
CD63-RFP
Overlay
B
Nuc
CD47-Ecto-tVSVG-GFP
Overlay
TLI
CD63-RFP
C
I
hTransferrin-tVSVG-GFP
D
I
hTransferrin-tVSVG-GFP
Nuc
Nuc
Figure S2. Expression and co-localization of genetic modifiers with exosome markers of CD63 and XPACK in cultured human 293T cells. Cells were co-transfected with either CD47-GFP and CD63-RFP (A), or CD47-Ecto-tVSVG-GFP and CD63-RFP (B), or hTransferrine-tVSVG-GFP and CD63-RFP (C), or NCAM-tVSVG-GFP and XPACK-RFP (D). The confocal images were recorded after 24 hrs of transfection for green fluorescence signals (column 1), red fluorescence signals (column 2), phase (TLI, column 3) and overlay (column 4), indicating co-localization of either CD47-GFP, CD47-Ecto-tVSVG-GFP, hTransferrin (green; pointed by white arrows) with exosome marker CD63 (green; white arrow) or XPACK (red; white arrows). The convergence of red and green fluorescence signals is indicated by yellow signals in the overly images (right column). The margin of nucleus (Nuc) is marked by dotted lines. Scale bar: 10 μm.
CD47-GFP
+
CD63-RFP
CD47-Ecto-tVSVG-GFP
+
CD63-RFP
hTransferrin-tVSVG-GFP
+
CD63-RFP
hTransferrin-tVSVG-GFP
+
XPACK-RFP

## Slide 3
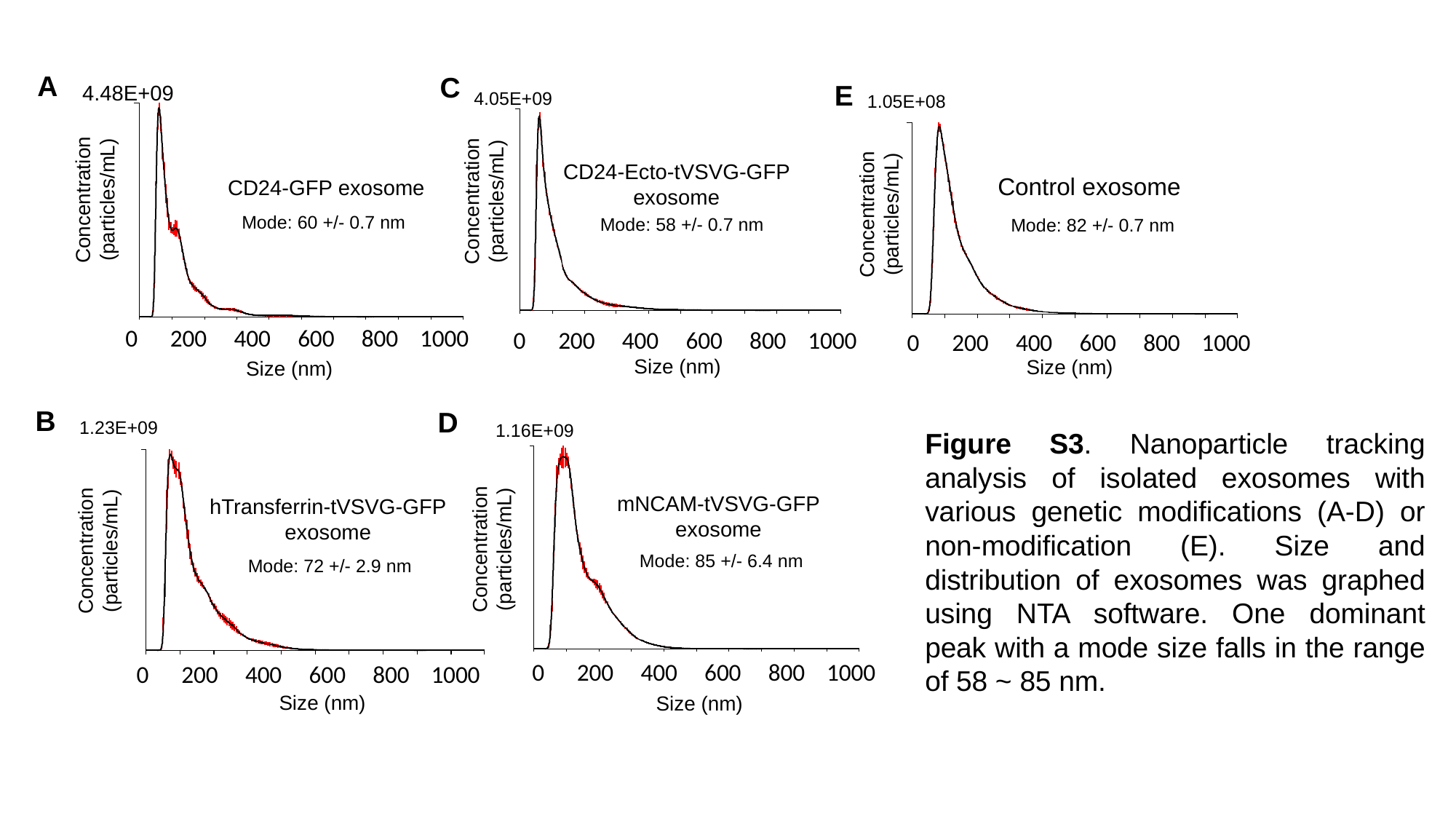

A
C
E
4.48E+09
4.05E+09
1.05E+08
CD24-Ecto-tVSVG-GFP exosome
Control exosome
CD24-GFP exosome
Concentration (particles/mL)
Concentration (particles/mL)
Concentration (particles/mL)
Mode: 60 +/- 0.7 nm
Mode: 58 +/- 0.7 nm
Mode: 82 +/- 0.7 nm
0 200 400 600 800 1000
0 200 400 600 800 1000
0 200 400 600 800 1000
Size (nm)
Size (nm)
Size (nm)
B
D
1.23E+09
1.16E+09
Figure S3. Nanoparticle tracking analysis of isolated exosomes with various genetic modifications (A-D) or non-modification (E). Size and distribution of exosomes was graphed using NTA software. One dominant peak with a mode size falls in the range of 58 ~ 85 nm.
mNCAM-tVSVG-GFP exosome
hTransferrin-tVSVG-GFP exosome
Concentration (particles/mL)
Concentration (particles/mL)
Mode: 85 +/- 6.4 nm
Mode: 72 +/- 2.9 nm
0 200 400 600 800 1000
0 200 400 600 800 1000
Size (nm)
Size (nm)
